# Co-expression Analysis of Sirtuins and Related Metabolic Biomarkers in Juveniles of Gilthead Sea Bream (*Sparus aurata*) With Differences in Growth Performance

**DOI:** 10.3389/fphys.2018.00608

**Published:** 2018-06-05

**Authors:** Paula Simó-Mirabet, Erick Perera, Josep A. Calduch-Giner, Juan M. Afonso, Jaume Pérez-Sánchez

**Affiliations:** ^1^Nutrigenomics and Fish Growth Endocrinology, Institute of Aquaculture Torre de la Sal-CSIC, Castellón, Spain; ^2^Aquaculture Research Group, Institute of Sustainable Aquaculture and Marine Ecosystems (IU-ECOAQUA), University of Las Palmas de Gran Canaria (GIA), Las Palmas, Spain

**Keywords:** fish, feed efficiency, lean phenotype, elongase 5, delta 9-desaturase, triacylglycerol lipase, lipoprotein lipase, immunoglobulin T

## Abstract

Sirtuins (SIRTs) represent a conserved protein family of deacetylases that act as master regulators of metabolism, but little is known about their roles in fish and livestock animals in general. The present study aimed to assess the value of SIRTs for the metabolic phenotyping of fish by assessing their co-expression with a wide-representation of markers of energy and lipid metabolism and intestinal function and health in two genetically different gilthead sea bream strains with differences in growth performance. Fish from the fast-growing strain exhibited higher feed intake, feed efficiency and plasma IGF-I levels, along with higher hepatosomatic index and lower mesenteric fat (lean phenotype). These observations suggest differences in tissue energy partitioning with an increased flux of fatty acids from adipose tissue toward the liver. The resulting increased risk of hepatic steatosis may be counteracted in the liver by reduced lipogenesis and enhanced triglyceride catabolism, in combination with a higher and more efficient oxidative metabolism in white skeletal muscle. These effects were supported by co-regulated changes in the expression profile of SIRTs (liver, *sirt1;* skeletal muscle, *sirt2;* adipose tissue, *sirt5-6*) and markers of oxidative metabolism (*pgc1*α*, cpt1a, cs, nd2, cox1*), mitochondrial respiration uncoupling (*ucp3*) and fatty acid and triglyceride metabolism (*ppar*α*, ppar*γ*, elovl5, scd1a, lpl, atgl*) that were specific to each strain and tissue. The anterior intestine of the fast-growing strain was better suited to cope with improved growth by increased expression of markers of nutrient absorption (*fabp2*), epithelial barrier integrity (*cdh1, cdh17*) and immunity (*il1*β, *cd8b, lgals1, lgals8, sIgT, mIgT*), which were correlated with low expression levels of *sirt4* and markers of fatty acid oxidation (*cpt1a*). In the posterior intestine, the fast-growing strain showed a consistent up-regulation of *sirt2, sirt3, sirt5* and *sirt7* concurrently with increased expression levels of markers of cell proliferation (*pcna*), oxidative metabolism (*nd2*) and immunity (*sIgT, mIgT*). Together, these findings indicate that SIRTs may play different roles in the regulation of metabolism, inflammatory tone and growth in farmed fish, arising as powerful biomarkers for a reliable metabolic phenotyping of fish at the tissue-specific level.

## Introduction

The capacity of aquaculture to meet the future demand for seafood will largely depend on the use of highly efficient domesticated animal stocks. Currently, less than 10% of the aquaculture production comes from genetically improved animals (Olesen et al., 2015); however, different selective breeding programs are in progress for most farmed European fish species, including the gilthead sea bream (*Sparus aurata* L.), a highly cultured perciform fish in all the Mediterranean basin. The main trait goals for gilthead sea bream breeding companies are growth performance, morphology, disease resistance and product quality with expected improvements in growth performance of 10–15% per generation (Gjedrem and Baranski, 2009; Janssen et al., 2015). However, the application of genomic tools in aquaculture is in its infancy (McAndrew and Napier, 2011), and few gilthead sea bream companies are currently using marker-assisted selection (MAS) (Janssen et al., 2015). The identification of new candidate genes for MAS, particularly for productive traits that are not easy to measure (e.g., feed efficiency, redox homeostasis, intestinal health), can be fueled using wide or targeted transcriptomic approaches (Chen et al., 2011; Cardoso et al., 2014; Choi et al., 2015). The interplay between nutrition and immune system is well recognized; however, the true integration of research on fish nutrition, growth, chronobiology, energy status, immune function and intestinal health is still far from clear despite recent and important advances in this field (Calduch-Giner et al., 2016; Estensoro et al., 2016; Martin and Król, 2017; Piazzon et al., 2017; Yúfera et al., 2017).

Fish exposed to sub-optimal rearing conditions are hampered with respect to health and growth, and genes known as master regulators of energy sensing are of special relevance for disclosing these types of metabolic disturbances. Most organisms have evolved to efficiently transition between anabolic and catabolic states, allowing them to survive in an environment in which nutrient availability is variable (Houtkooper et al., 2012; Laplante and Sabatini, 2012). Nutrient stress is generally considered from the standpoint of how cells detect and respond to an insufficient supply of nutrients (Wellen and Thompson, 2010). However, cells and organisms also experience stress with nutrient excess as a major readout of nutrient uptake is the level of reactive oxygen species (ROS) produced by mitochondria (Wellen and Thompson, 2010), which limit voluntary feed intake (Saravanan et al., 2012) and growth (Fernández-Díaz et al., 2006; Rise et al., 2015) in farmed fish. Different mechanisms operate within cells to balance ROS production and scavenging to keep ROS within physiological levels. The mitochondrial uncoupling proteins (UCPs) act as a highly conserved safety valve that activates futile cycles of energy to alleviate ROS production (Mailloux and Harper, 2011). These cycles become rapidly inactive when the oxidative capacity of the tissue is improved, or the supply of metabolic fuels does not exceed the tissue energy demand in a wide range of experimental models, including fish (Nabben and Hoeks, 2008; Bermejo-Nogales et al., 2011, 2014). Moreover, the antioxidant defense system relies mostly on superoxide dismutase, glutathione peroxidase, glutathione reductase, thioredoxin, thioredoxin reductase and catalase, operating as ROS scavengers (Martínez-Álvarez et al., 2005; Pacitti et al., 2014). As part of this complex regulatory system, nutrient and energy availability are sensed at multiple levels. AMP-activated protein kinase (AMPK) inhibits proliferation and growth in response to ATP depletion, while the mammalian target of rapamycin (mTOR) is activated by nutrients and signaling growth factors to promote mitochondrial metabolism, protein synthesis and cell growth (Wellen and Thompson, 2010; Laplante and Sabatini, 2012). In addition, protein post-translational modifications such as O-GlcNAcylation, glycosylation and acetylation/deacetylation play key roles in the adaptation to metabolic stress produced by elevated levels of intracellular metabolites, including ROS (Wellen and Thompson, 2010).

Among protein post-translational modifications, deacetylation is particularly sensitive to metabolic states through the action of deacetylases, first represented by NAD^+^-dependent sirtuin deacetylases/deacylases/ADP-ribosyltransferases (SIRTs). Most SIRTs couple protein deacetylation of histone and non-histone substrates with the energy status of the cell via the cellular NAD^+^/NADH ratio (Schwer and Verdin, 2008; Houtkooper et al., 2012; Schmeisser et al., 2013; Masri, 2015). Proteomic studies following the initial discovery of histone acetylation have also revealed that thousands of proteins are abundantly acetylated (Zhao et al., 2010; Guan and Xiong, 2011; Choudhary et al., 2014), and their deacetylation commonly leads to increased stability and catalytic activity in the case of metabolic enzymes (Verdin et al., 2010; Houtkooper et al., 2012). In contrast, deacetylation of histones is an epigenetic mechanism associated with repression of gene expression (Lundby et al., 2012). These mechanisms yield a highly regulated proteome with key roles played by SIRTs, at both the transcriptional and post-translational levels, in the maintenance of energy homeostasis (Schwer and Verdin, 2008; Zhao et al., 2010; Houtkooper et al., 2012) as well as in muscle growth through negative regulation of IGF-I and mTOR signaling (Ghosh et al., 2010; Sharples et al., 2015). Accordingly with this central role in metabolism regulation, SIRTs are virtually ubiquitous throughout all kingdoms of life, ranging in abundance from one type in bacteria to seven types in vertebrates (Greiss and Gartner, 2009). This feature offers the possibility of complementary but also non-redundant and tissue-specific energy sensing mechanisms, which is reflected by the different cellular locations of different SIRTs. SIRT1, SIRT6, and SIRT7 generally reside in the nucleus; SIRT2 is primarily cytosolic, although it is shuttled to the nucleus during the G2/M transition of the cell cycle (Gomes et al., 2015); and SIRT3-5 are mitochondrial proteins (Jing and Lin, 2015).

Most research on SIRTs has been carried out in humans and rodents, which limits our understanding of the evolution of SIRT regulation and function. However, the seven SIRT counterparts of higher vertebrates have been molecularly characterized in gilthead sea bream (Simó-Mirabet et al., 2017a). The sequence analysis of these counterparts has revealed a strict conservation of the characteristic catalytic domain, and phylogenetic analysis has revealed three major clades corresponding to SIRT1-3, SIRT4-5, and SIRT6-7 that reflect the accepted classification of SIRTs (Frye, 2000). Gene expression profiling has also demonstrated that the molecular signatures of fish SIRTs in gilthead sea bream are strongly influenced by nutrient availability and tissue-specific metabolic capabilities (Simó-Mirabet et al., 2017a). In this scenario, changes in the SIRT gene expression pattern contribute to triggering the metabolic switch from adipogenesis to lipolysis with the increased demand of metabolic fuels by peripheral tissues during fasting or caloric restriction. Other studies in fish have related SIRTs to ammonia levels (Connon et al., 2011), cold exposure (Teigen et al., 2015), spatial learning (Rajan et al., 2015), changes in blood glucose (Otero-Rodiño et al., 2016) and adipocyte maturation during hypoxia (Ekambaram and Parasuraman, 2017). Nevertheless, SIRT function and regulation remain poorly studied in fish and in livestock animals in general (Ghinis-Hozumi et al., 2013).

The present study aimed to assess the gene expression pattern of SIRTs in fish under non-restricted feeding and the value of their gene expression profile as a new tool for metabolic phenotyping of farmed fish. This was accomplished by measuring SIRTs' co-regulated expression with markers of intermediary metabolism, immunological status, and intestine function and integrity in two gilthead sea bream strains with known differences in growth performance. The rationale of the study was that differences in key performance indicators necessarily reflect different uses of nutrients and energy, being voluntary feed intake and growth limited by the capacity of fish to preserve redox balance (Saravanan et al., 2012; Rise et al., 2015; Danzmann et al., 2016). Accordingly, the working hypothesis was that fish with higher growth rates would be able to grow efficiently in a cellular milieu with an enhanced risk of oxidative stress, contributing the differential regulation of SIRTs to readjust and preserve metabolic homeostasis at each tissue.

## Material and methods

### Fish

The gilthead sea bream is highly cultured in Europe, with several hatcheries operating mostly, but not exclusively, in the Mediterranean basin (Janssen et al., 2015). In the present study, we used fish from two geographically distant hatcheries (henceforth called strains 1 and 2), which have regularly performed differently under the same growout conditions in our experimental facilities.

For the genotyping of these two populations, thirty fish of each strain were characterized for specific microsatellite markers. Both fish populations were recognized as genetically different, with 3.5% of genetic differentiation (Fst = 0.0351), by SMsa1 multiplex PCR of 10 loci (A5, C3, C12, D4, E1, E4, F6, I9, L11, M5) (Lee-Montero et al., 2013) (Supplementary Table 1). Briefly, DNA was extracted from the fin by using the BioSprint 96 DNA Blood Kit (QIAGEN®) operated by a Biosprint 96 robot. The concentration of extracted DNA was measured by using a NanoDrop 8000 spectrophotometer v.3.7 (Thermo Fisher Scientific) and normalized to 80 ng/μl prior to PCR amplification. PCR reactions were carried out by using a TECAN robot Freedom Evo (Tecan Schweiz AG, Switzerland), and Freedom Evowar® Standard v.2.5 software following the manufacturer's instructions. Genotypes were estimated by GENEMAPPER v.3.7 software using the SMsa1-kit created by Lee-Montero et al. (2013).

### Feeding trial

Gilthead sea bream juveniles of strain 1 and strain 2 were acclimatized for 6 weeks to the indoor experimental facilities of the Institute of Aquaculture Torre de la Sal (IATS-CSIC). Then, 13-15 g fish from both strains were distributed among 90 L tanks in triplicate groups of 25 fish each. The trial was conducted under natural photoperiod and temperature conditions at the latitude of the IATS (40°5 N; 0°10E) from May to July (8 weeks), increasing the water temperature from 20° to 25°C. The oxygen content of water was consistently higher than 75% saturation, and unionized ammonia remained below toxic levels (<0.02 mg/L). Fish were fed with a standard diet (EFICO YM 568; BioMar, Spain) twice a day until visual satiety. At the end of the trial, 12 fish per strain (four randomly selected fish per tank) were anesthetized with 3-aminobenzoic acid ethyl ester (MS-222, 100 μg/mL) and blood was quickly taken from caudal vessels with heparinized syringes. One aliquot was used for hemoglobin measurements. The remaining blood was centrifuged at 3,000 × g for 20 min at 4°C, and the plasma was stored at −80°C until biochemical assays. Liver, white skeletal muscle, adipose tissue, and anterior and posterior intestine sections were rapidly excised from 12 fish per strain, frozen in liquid nitrogen and stored at −80°C until RNA extraction.

All procedures were carried out according to present IATS-CSIC Review Board and European (2010/63/EU) animal directives and Spanish laws (Royal Decree RD53/2013) on the handling of experimental animals.

### Blood biochemistry

Hemoglobin (Hb) concentration was determined using a HemoCue B-Hemoglobin Analyser® (AB, Leo Diagnostic, Sweden). Plasma glucose was measured by the glucose oxidase method (Thermo Fisher Scientific, Waltham, Massachusetts, USA). Total plasma cholesterol was determined using cholesterol esterase/cholesterol dehydrogenase reagent (Thermo Fisher Scientific). Plasma soluble proteins were measured with the Bio-Rad protein reagent (Hercules, California, USA), with bovine serum albumin as a standard. Plasma growth hormone (GH) was determined by a homologous gilthead sea bream radioimmunoassay (RIA) as previously detailed (Martínez-Barberá et al., 1995). The sensitivity and midrange (ED50) of the GH RIA assay were 0.15 and 1.8 ng/mL, respectively. Plasma insulin-like growth factors (IGFs) were extracted by acid-ethanol cryoprecipitation (Shimizu et al., 2000), and the concentration of IGF-I was measured by a generic fish IGF-I RIA validated for several Mediterranean perciform fish (de Celis et al., 2004). The sensitivity and midrange of the IGF-I RIA assay were 0.05 and 0.7−0.8 ng/mL, respectively. Plasma cortisol levels were analyzed using an EIA kit (kit RE52061, IBL, International GmbH, Germany). The detection limit of the cortisol assay was 50 pg/mL, with a midrange of 700 pg/mL. All commercial kits were used according to the manufacturers' instructions.

### Gene expression profiling

RNA was extracted using the MagMAX-96 total RNA isolation kit (Life Technologies, Carlsbad, CA, USA). RNA yield was 50–100 μg, with 260:280 nm absorbance ratios (A260/280) of 1.9-2.1. RNA integrity number (RIN) values of 8–10 (Agilent 2100 Bioanalyzer) were indicative of clean and intact RNA. Reverse transcription (RT) of 500 ng total RNA was performed with random decamers using a High-Capacity cDNA Archive Kit (Applied Biosystems, Foster City, CA, USA). Negative control reactions were run without reverse transcriptase. Two different 96-well PCR-arrays of 28–39 markers of metabolic and intestinal health condition were designed for the simultaneous gene expression profiling of liver/white skeletal muscle/adipose tissue and intestine, respectively (Table 1). A housekeeping gene (β-Actin) and controls of PCR performance were included in each array. Briefly, 660 pg of total cDNA was used in 25 μL PCR reactions. PCR wells contained 2x SYBR Green Master Mix (Bio-Rad, Hercules, CA, USA) and specific primers at a final concentration of 0.9 μM (Supplementary Table 2 and 3). All pipetting operations for the PCR-arrays were performed by an EpMotion 5070 Liquid Handling Robot (Eppendorf, Hamburg, Germany) to improve data reproducibility. Real-time quantitative PCR was carried out in an Eppendorf Mastercycler Ep Realplex (Eppendorf, Germany). The PCR amplification program consisted of an initial denaturation step at 95°C for 3 min, followed by 40 cycles of denaturation for 15 s at 95°C and annealing/extension for 60 s at 60°C. The efficiency of the PCR reactions was consistently higher than 90% and similar among all the genes. The specificity of the reactions was verified by melting curve analysis (ramping rates of 0.5°C/10 s over a temperature range of 55–95°C). Negative controls without a template were routinely performed for each primer set. Gene expression was calculated using the delta-delta Ct method (Livak and Schmittgen, 2001). For multi-gene analysis, all values for a given tissue were referenced to the expression level of *sirt1* in strain 1 fish, for which a value of 1 was arbitrarily assigned. Fold-changes in gene expression were calculated as the expression ratio between strain 1 and strain 2. A value > 1 indicates higher expression levels in strain 1, and values <1 indicate lower expression levels in strain 1.

**Table 1:** Genes included in the intestine (†) and liver/adipose/muscle (^*^) tissue pathway-focused PCR arrays.

**Gene name/category**	**Symbol**
**ENERGY SENSING**	
Sirtuin 1	sirt1^*^^†^
Sirtuin 2	sirt2^*^^†^
Sirtuin 3	sirt3^*^^†^
Sirtuin 4	sirt4^*^^†^
Sirtuin 5	sirt5^*^^†^
Sirtuin 6	sirt6^*^^†^
Sirtuin 7	sirt7^*^^†^
**OXIDATIVE METABOLISM**	
Proliferator-activated receptor gamma coactivator 1 alpha	pgc1α^*^^†^
Carnitine palmitoyltransferase 1A	cpt1a^*^^†^
Citrate synthase	cs^*^^†^
NADH-ubiquinone oxidoreductase chain 2	nd2^*^^†^
Cytochrome c oxidase subunit I	cox1^*^^†^
**MITOCHONDRIAL RESPIRATION UNCOUPLING**	
Uncoupling protein 1	ucp1^*^^†^
Uncoupling protein 2	ucp2^*^
Uncoupling protein 3	ucp3^*^
**LIPID METABOLISM**	
Peroxisome proliferator-activated receptor α	pparα^*^
Peroxisome proliferator-activated receptor γ	pparγ^*^
Elongation of very long chain fatty acids 4	elovl4^*^
Elongation of very long chain fatty acids 5	elovl5^*^
Elongation of very long chain fatty acids 6	elovl6^*^
Fatty acid desaturase 2	fads2^*^
Stearoyl-CoA desaturase 1a	scd1a^*^
Stearoyl-CoA desaturase 1b	scd1b^*^
Phosphatidylethanolamine N-methyltransferase	pemt^*^
Hepatic lipase	hl^*^
Lipoprotein lipase	lpl^*^
Hormone sensitive lipase	hsl^*^
Adipose triglyceride lipase	atgl^*^
**CELL DIFFERENTIATION AND PROLIFERATION**	
Proliferating cell nuclear antigen	*pcna*^†^
**INTESTINAL EPITHELIAL BARRIER**	
Occludin	*ocln*^†^
Claudin-15	*cldn15*^†^
Cadherin-1	*cdh1*^†^
Cadherin-17	*cdh17*^†^
**ENTEROCYTE MASS AND NUTRIENT ABSORPTION**	
Intestinal-type alkaline phosphatase	*alpi*^†^
Liver type fatty acid-binding protein	*fabp1*^†^
Intestinal fatty acid-binding protein	*fabp2*^†^
Ileal fatty acid-binding protein	*fabp6*^†^
**MUCUS PRODUCTION AND GOBLET CELL DIFFERENTIATION**	
Mucin 2	*muc2*^†^
Mucin 13	*muc13*^†^
Transcription factor HES-1-B	*hes1-b*^†^
**IMMUNOLOGICAL/INFLAMMATORY STATUS**	
Tumor necrosis factor-alpha	*tnfα*^†^
Interleukin-1 beta	*il1β*^†^
Interleukin-6	*il6*^†^
Interleukin-8	*il8*^†^
Interleukin-10	*il10*^†^
CD4	*cd4*^†^
CD8 alpha	*cd8a*^†^
CD8 beta	*cd8b*^†^
Galectin-1	*lgals1*^†^
Galectin-8	*lgals8*^†^
Secreted immunoglobulin M	*sIgM*^†^
Secreted immunoglobulin T	*sIgT*^†^
Membrane immunoglobulin M	*mIgM*^†^
Membrane immunoglobulin T	*mIgT*^†^

### Statistical analyses

Data pertaining to growth performance, blood biochemistry and gene expression of the two fish strains in liver, white skeletal muscle and adipose tissue were analyzed by Student's *t*-test. Two-way analysis of variance (ANOVA) was carried out to analyze intestinal gene expression, with both the intestine segment and the fish strain as sources of variation. The significance level was set to *P* < 0.05 in all tests performed. These analyses were conducted using SigmaPlot version 13.0 (Systat Software, San Jose, CA).

To confirm the genetic differentiation between both fish strain used, the following log-linear model was used in SPSS software (IBM Corp., Armonk, N.Y., USA) for statistical analysis when both populations were compared for different microsatellite markers (referred as factors A and B):

ln *f*_*ij*_ = μ + α_*i*_+ β_*j*_ + αβ_*ij*_, where

*f*_*ij*_ = is the expected frequency in row *i*, column *j* of the two-way contingency table
μ = is the mean of the logarithms of the expected frequencies
α_*i*_ = is the effect of category *i* of factor A
β_*j*_ = is the effect of category *j* of factor B
αβ_*ij*_ = is the interaction term indicating the dependence of category *i* of factor A on category *j* of factor B.

The genetic flow between populations was estimated through Fst (Nei, 1973), by using the GENEPOP software (Raymond and Rousset, 1995; Rousset, 2008).

## Results

### Growth performance and blood biochemistry

Data pertaining to growth performance and blood biochemistry of the two genetically different (3.5% of genetic differentiation) strains are shown in Table 2. Fish of strain 1 showed higher feed intake and grew faster than fish of strain 2 with specific growth rates of 2.1 and 1.6, respectively. Feed efficiency (FE) was also significantly improved (1.2-fold higher) in fish of strain 1. Organosomatic indexes were determined for viscera, liver and mesenteric fat as tissue to body weight ratios. The resulting viscerosomatic (VSI) and mesenteric fat (MFI) indexes were significantly lower in fish of strain 1, whereas the opposite was observed for the hepatosomatic index (HSI). Regarding blood biochemistry, significant effects of fish strain on circulating levels of hemoglobin, glucose, GH and cortisol were not found. However, plasma levels of cholesterol, proteins and IGF-I were higher in strain 1 fish than in strain 2 fish.

**Table 2:** Growth performance and blood biochemistry of two different gilthead sea bream strains fed to satiety over the course of 8-weeks (May–July) under natural light and temperature conditions.

	**Strain 1**	**Strain 2**	***P*^a^**
Initial body weight (g)	15.1 ± 0.04	13.2 ± 0.03	< 0.001
Final body weight (g)	50.5 ± 0.60	32.3 ± 0.03	< 0.001
Feed intake (g DM/fish)	42.1 ± 0.20	27.5 ± 0.70	< 0.001
SGR (%)^b^	2.11 ± 0.02	1.57 ± 0.01	< 0.001
FE (%)^c^	0.84 ± 0.01	0.69 ± 0.02	0.006
Viscera (g)	4.95 ± 0.17	3.97 ± 0.22	0.002
Liver (g)	1.06 ± 0.15	0.68 ± 0.03	< 0.001
Mesenteric fat (g)	0.74 ± 0.07	0.91 ± 0.10	0.181
VSI (%)^d^	9.28 ± 0.18	10.1 ± 0.31	0.037
HSI (%)^e^	1.98 ± 0.07	1.76 ± 0.07	0.05
MFI (%)^f^	1.38 ± 0.12	2.29 ± 0.22	0.002
**BLOOD BIOCHEMISTRY**			
Hemoglobin (g/dL)	6.19 ± 0.11	5.90 ± 0.24	0.281
Glucose (mg/dL)	43.8 ± 1.47	44.2 ± 2.13	0.431
Total cholesterol (mg/dL)	148.2 ± 8.46	100.2 ± 20.50	0.001
Total proteins (g/L)	45.4 ± 1.41	39.0 ± 0.86	< 0.001
GH (ng/mL)	4.90 ± 2.10	6.85 ± 2.06	0.130
IGF-I (ng/mL)	57.5 ± 4.12	28.8 ± 3.88	< 0.001
Cortisol (ng/mL)	12.2 ± 3.01	14.1 ± 7.40	0.788

*Data on body weight, feed intake, and growth indices are the mean ± SEM of triplicate tanks (25 fish/tank). Data on blood biochemistry, viscera, liver and mesenteric fat weights are the mean ± SEM of 12 fish (4 fish/tank; 12 fish/strain)*.

a *Result values from t-test*.

b *Specific growth rate = 100 × (ln final body weight-ln initial body weight/days)*.

c *Feed efficiency = weight gain/dry feed intake*.

d *Viscerosomatix index = (100 × viscera weight)/fish weight*.

e *Hepatosomatic index = (100 × liver weight)/fish weight*.

f *Mesenteric fat index = (100 × mesenteric fat weight)/fish weight*.

### Transcriptional profiling of liver, adipose tissue, and skeletal muscle

**Figure 1:**
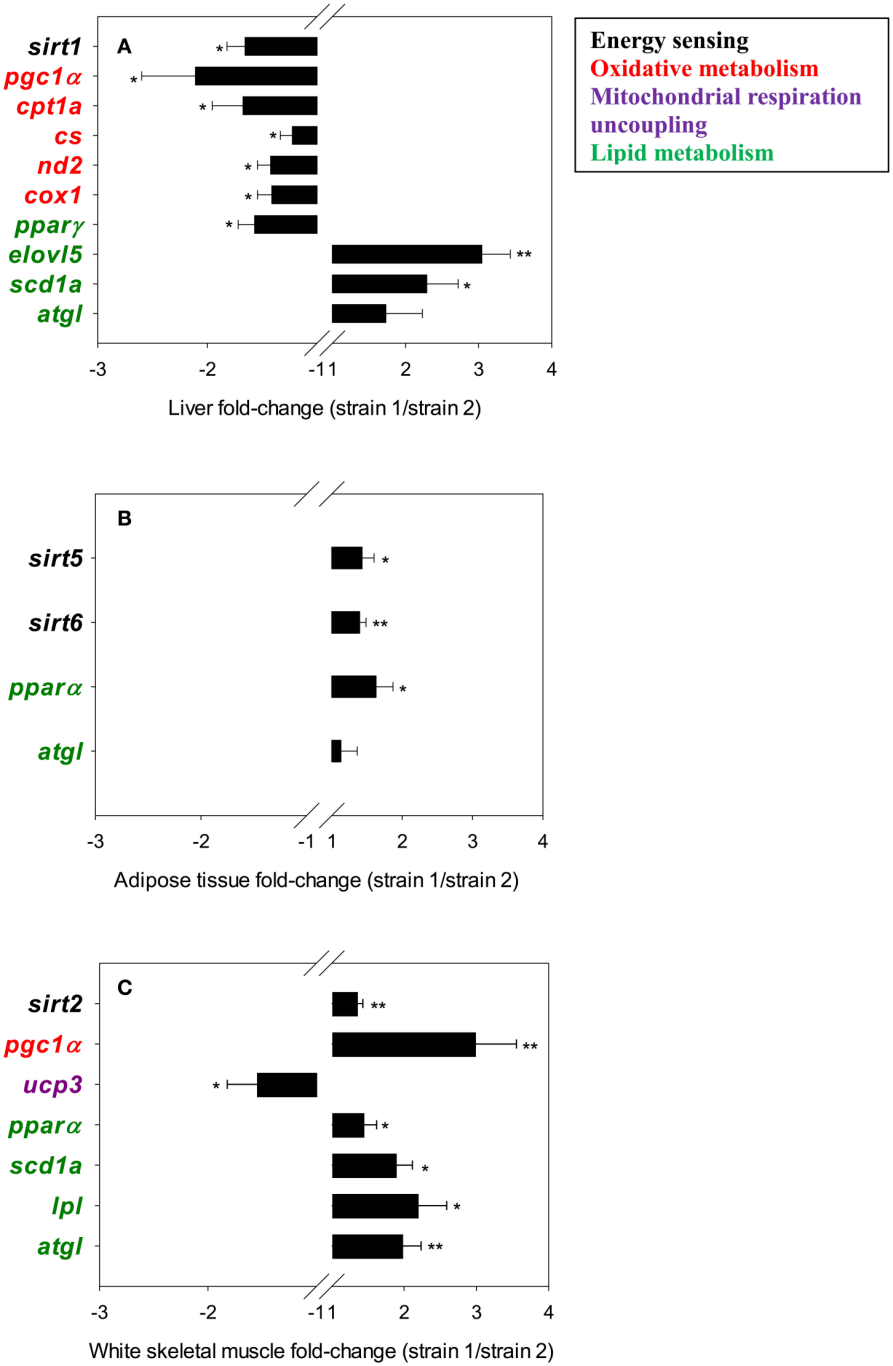
Fold-changes (strain 1/strain 2) of differentially expressed genes in liver tissue **(A)**, adipose tissue **(B)** and white skeletal muscle **(C)**. The asterisks indicate statistically significant differences (^*^P < 0.05, ^**^P < 0.01) between strains. Values >1 indicate up-regulated genes in fish of strain 1; values < 1 indicate down-regulated genes in fish strain 1.

Data regarding relative gene expression in liver, adipose and white skeletal muscle tissue are shown in Table 3. To simplify the visualization of the results, only fold- changes (calculated as the ratio strain 1/strain 2) of differentially expressed genes are represented in Figure 1. The exception is *atgl*, which is included in the graphical representation of all tissues, although it's overall increased expression in fish of strain 1 was only statistically significant in skeletal muscle.

**Table 3:** Relative mRNA expression in liver, adipose tissue (AT) and white skeletal muscle (WSM) of selected markers of intermediary metabolism in two different gilthead sea bream strains.

	**Liver**			**AT**			**WSM**		
	**Strain 1**	**Strain 2**	***P*-value**	**Strain 1**	**Strain 2**	***P*-value**	**Strain 1**	**Strain 2**	***P*-value**
*sirt1*	1.02 ± 0.11	1.69 ± 0.17	**0.011**	1.02 ± 0.09	0.86 ± 0.03	0.150	1.03 ± 0.11	1.12 ± 0.10	0.566
*sirt2*	2.52 ± 0.25	2.71 ± 0.11	0.508	0.82 ± 0.16	0.60 ± 0.07	0.246	1.90 ± 0.11	1.41 ± 0.05	**0.002**
*sirt3*	0.37 ± 0.05	0.36 ± 0.03	0.835	0.44 ± 0.04	0.42 ± 0.02	0.661	0.16 ± 0.02	0.17 ± 0.01	0.641
*sirt4*	0.20 ± 0.05	0.21 ± 0.03	0.983	0.08 ± 0.02	0.07 ± 0.01	0.530	0.15 ± 0.02	0.14 ± 0.01	0.764
*sirt5*	2.36 ± 0.12	2.39 ± 0.16	0.886	0.76 ± 0.09	0.53 ± 0.02	**0.048**	2.22 ± 0.27	2.18 ± 0.12	0.884
*sirt6*	0.25 ± 0.04	0.28 ± 0.02	0.214	0.28 ± 0.02	0.20 ± 0.01	**0.002**	0.13 ± 0.02	0.14 ± 0.01	0.660
*sirt7*	0.63 ± 0.07	0.63 ± 0.03	0.469	0.32 ± 0.05	0.24 ± 0.02	0.177	0.43 ± 0.05	0.42 ± 0.03	0.911
*pgc1α*	0.27 ± 0.06	0.56 ± 0.09	**0.034**	0.02 ± 0.01	0.01 ± 0.002	0.356	1.00 ± 0.19	0.34 ± 0.06	**0.006**
*cpt1a*	4.52 ± 0.72	7.57 ± 0.76	**0.016**	2.08 ± 0.21	2.14 ± 0.07	0.818	9.43 ± 1.23	10.2 ± 1.18	0.666
*cs*	7.32 ± 0.63	8.98 ± 0.25	**0.028**	6.21 ± 0.75	5.28 ± 0.45	0.358	59.9 ± 3.10	60.3 ± 2.69	0.918
*nd2*	306.9 ± 25.8	436.9 ± 47.7	**0.038**	75.8 ± 8.96	63.2 ± 4.19	0.266	248.8 ± 33.9	233.3 ± 14.4	0.682
*cox1*	593.4 ± 57.8	838.4 ± 57.9	**0.016**	225.1 ± 27.2	167.1 ± 9.3	0.133	1028.3 ± 198.9	918.5 ± 53.8	0.606
*ucp1*	100.6 ± 7.8	113.2 ± 13.0	0.410	–	–	–	–	–	–
*ucp2*	0.02 ± 0.003	0.02 ± 0.003	0.871	0.05 ± 0.01	0.06 ± 0.01	0.566	0.80 ± 0.14	0.81 ± 0.13	0.980
*ucp3*	–	–	–	–	–	–	13.4 ± 2.20	20.8 ± 1.59	**0.026**
*pparα*	29.1 ± 3.73	31.3 ± 3.27	0.661	1.23 ± 0.18	0.75 ± 0.08	**0.045**	3.52 ± 0.34	2.45 ± 0.19	**0.044**
*pparγ*	4.14 ± 0.42	6.51 ± 0.92	**0.047**	11.98 ± 0.76	15.2 ± 3.72	0.422	0.86 ± 0.14	0.80 ± 0.06	0.699
*elovl4*	4.21 ± 0.92	5.56 ± 0.26	0.190	0.13 ± 0.03	0.10 ± 0.02	0.423	0.28 ± 0.02	0.38 ± 0.06	0.161
*elovl5*	37.7 ± 4.85	12.4 ± 3.48	**0.002**	0.64 ± 0.11	0.49 ± 0.06	0.225	0.69 ± 0.30	0.55 ± 0.06	0.635
*elovl6*	26.1 ± 5.05	21.2 ± 5.71	0.527	0.73 ± 0.13	0.54 ± 0.11	0.273	0.32 ± 0.04	0.35 ± 0.04	0.545
*fads2*	92.8 ± 17.7	88.1 ± 4.98	0.799	0.57 ± 0.18	0.39 ± 0.19	0.491	0.11 ± 0.01	0.15 ± 0.03	0.282
*scd1a*	12.8 ± 2.40	5.58 ± 1.13	**0.027**	1.31 ± 0.25	1.04 ± 0.32	0.512	1.91 ± 0.24	1.01 ± 0.26	**0.032**
*scd1b*	32.6 ± 9.2	36.5 ± 19.5	0.863	50.12 ± 10.17	50.3 ± 15.7	0.991	4.22 ± 1.02	2.30 ± 0.38	0.110
*pemt*	6.50 ± 0.49	6.98 ± 0.61	0.545	0.17 ± 0.05	0.15 ± 0.01	0.724	0.41 ± 0.05	0.36 ± 0.03	0.371
*hl*	178.9 ± 44.7	184.9 ± 12.1	0.900	–	–	–	–	–	–
*lpl*	49.2 ± 19.2	51.0 ± 6.80	0.932	99.42 ± 14.8	90.9 ± 27.6	0.345	5.04 ± 1.30	2.31 ± 0.29	**0.020**
*hsl*	2.94 ± 0.64	3.64 ± 0.31	0.348	10.54 ± 1.23	13.9 ± 2.17	0.205	1.19 ± 0.34	0.89 ± 0.04	0.358
*atgl*	2.38 ± 0.68	1.38 ± 0.25	0.150	1.21 ± 0.25	1.07 ± 0.18	0.647	0.73 ± 0.10	0.37 ± 0.03	**0.004**

*Values are the mean ± SEM of 6 fish. P-values are the result of Student t-test between strains in a given tissue. Bold values indicate statistically significant differences (P < 0.05). All data values for each tissue were in reference to the expression level of sirt1 of strain 1 with an arbitrary assigned value of 1. Colors correspond to different gene categories defined in Table 1*.

In liver (Figure 1A), *sirt1* and mitochondrial genes related to oxidative metabolism, including markers of mitochondrial biogenesis and glucose/fatty acid (FA) metabolism (*pgc1*α), tricarboxylic acid (TCA) cycle (*cs*), oxidative phosphorylation (OXPHOS; *nd2, cox1*), mitochondrial FA transport and β-oxidation (*cpt1a*), were expressed at a lower rate in fish of strain 1. The expression of the lipogenic *ppar*γ was also lower in fish of strain 1. Conversely, the FA elongase *elovl5* and the delta 9-desaturase *scd1a* exhibited higher expression levels in the liver of fish of strain 1. The same trend was observed for the intracellular triacylglycerol lipase *atgl*, although the observed changes were not statistically significant. In adipose tissue (Figure 1B), *sirt5, sirt6*, and *ppar*α exhibited higher expression rates in strain 1 fish than in strain 2 fish. The same trend was observed for *atgl*, although it was not statistically significant. In the white skeletal muscle (Figure 1C), higher *sirt2* transcript abundance in fish of strain 1 occurred along with higher gene expression levels of key genes of mitochondrial biogenesis (*pgc1*α), tissue FA uptake (*lpl*), lipid catabolism (*atgl, ppar*α) and metabolism of monounsaturated FAs (*scd1a*). Conversely, the expression rate of the mitochondrial respiration uncoupling protein of skeletal muscle tissues (*ucp3*) was lower in fish of strain 1.

### Transcriptional profiling of intestine

**Figure 2:**
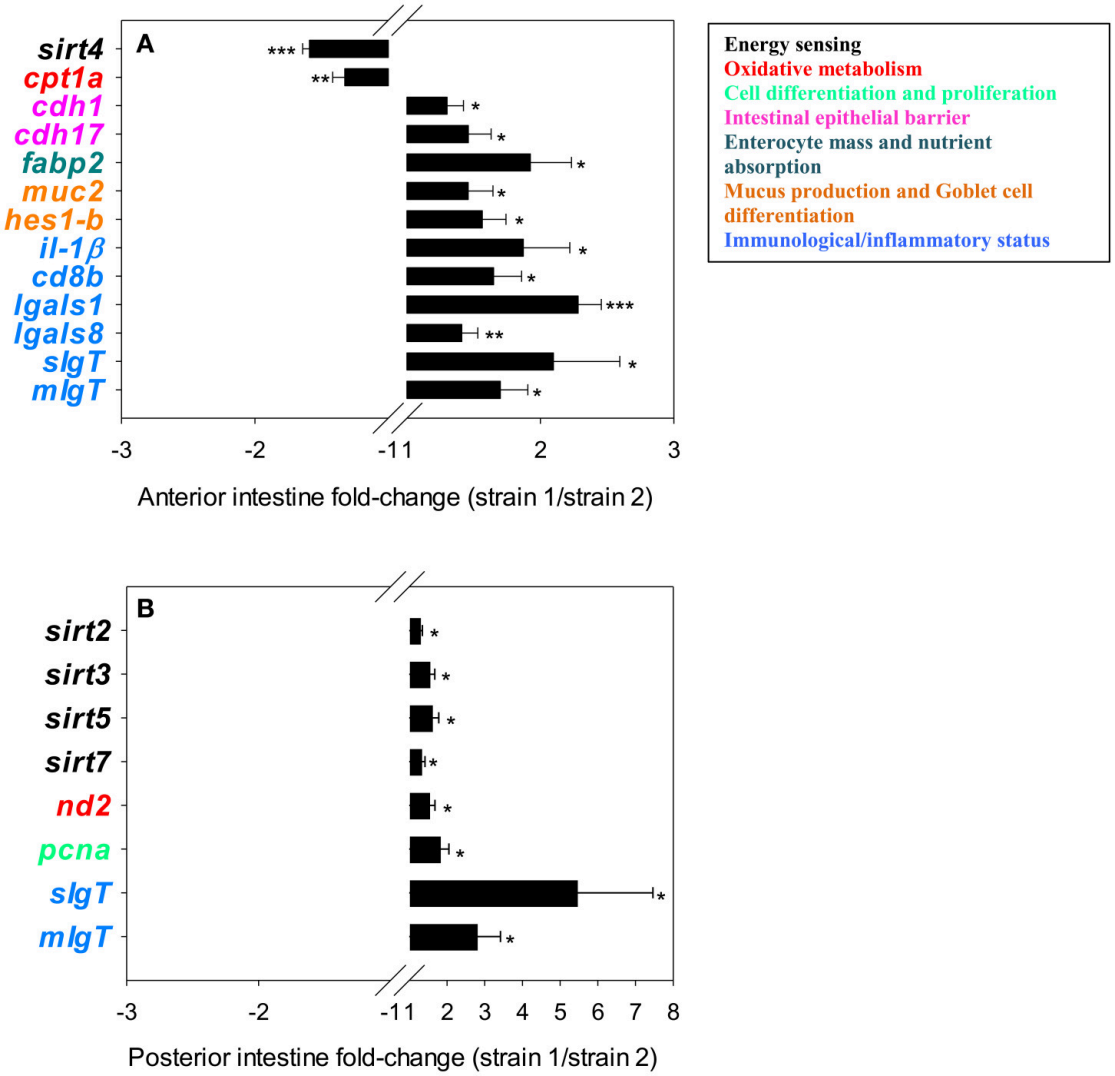
Fold-changes (strain 1/strain 2) of differentially expressed genes in the anterior **(A)** and posterior **(B)** intestinal segments. The asterisks indicate statistically significant differences (^*^P < 0.05, ^**^P < 0.01, ^***^P < 0.001) between strains. Values >1 indicate up-regulated genes in fish of strain 1; values <1 indicate down-regulated genes in fish strain 1.

Results regarding the intestinal expression of genes related to intermediary metabolism and intestine function and integrity are shown in Table 4. The two-way ANOVA indicated that most genes included in the intestine PCR-array were spatially regulated, with 30 genes out of 39 being differentially expressed along the intestine. A fish strain effect was also observed for 19 genes in at least one intestine segment. In the anterior intestine (Figure 2A), the expression of *sirt4* and *cpt1a* was lower in strain 1 fish, but the opposite trend was found for the other differentially expressed genes, including markers of enterocyte mass and intracellular FA transport (*fabp2*), epithelial barrier integrity (*cdh1, cdh17*), mucus production and Goblet cell differentiation (*muc2, hes1-b*) and immunological/inflammatory status (*il1*β, *cd8b, lgals1, lgals8, sIgT, mIgT*). In the posterior intestine (Figure 2B), the transcript abundance of different SIRTs (*sirt2, sirt3, sirt5, sirt7*) was significantly higher in fish of strain 1. This higher abundance occurred in combination with increased expression of markers of OXPHOS (*nd2*), cell proliferation (*pcna*) and immunity (*sIgT, mIgT*).

**Table 4:** Relative mRNA expression of selected markers of intermediary metabolism and intestine function and integrity in two different gilthead sea bream strains.

	**Anterior intestine**		**Posterior intestine**		***P*****-value**	
	**Strain 1**	**Strain 2**	**Strain 1**	**Strain 2**	**Int. section**	**Strain**	**Interaction**
*sirt1*	1.02 ± 0.09	0.97 ± 0.08	1.30 ± 0.09	1.12 ± 0.06	**0.019**	0.174	0.432
*sirt2*	1.17 ± 0.06	1.01 ± 0.04	1.31 ± 0.07^*^	1.02 ± 0.07	0.233	**0.003**	0.301
*sirt3*	0.28 ± 0.02	0.28 ± 0.04	0.37 ± 0.03^*^	0.24 ± 0.03	0.453	**0.046**	0.061
*sirt4*	0.19 ± 0.01^***^	0.30 ± 0.03	0.14 ± 0.02	0.14 ± 0.01	**<0.001**	**<0.001**	**<0.001**
*sirt5*	0.87 ± 0.07	0.79 ± 0.07	1.24 ± 0.14^*^	0.77 ± 0.06	0.070	**0.008**	**0.047**
*sirt6*	0.17 ± 0.01	0.16 ± 0.01	0.21 ± 0.02	0.18 ± 0.01	**0.047**	0.148	0.635
*sirt7*	0.26 ± 0.01	0.26 ± 0.02	0.41 ± 0.03^*^	0.31 ± 0.01	**<0.001**	**0.013**	**0.018**
*pgc1α*	3.18 ± 0.19	3.24 ± 0.47	2.66 ± 0.17	2.23 ± 0.20	**0.007**	0.442	0.333
*cpt1a*	6.36 ± 0.40^**^	8.43 ± 0.32	4.82 ± 0.19	4.31 ± 0.45	**<0.001**	**0.037**	**0.002**
*cs*	33.11 ± 2.23	37.7 ± 1.65	15.3 ± 1.75	13.3 ± 0.90	**<0.001**	0.475	0.088
*nd2*	118.1 ± 6.11	116.7 ± 9.02	112.5 ± 11.7^*^	73.8 ± 5.35	**0.012**	**0.031**	**0.042**
*cox1*	279.5 ± 24.2	281.7 ± 22.6	463.9 ± 37.2	383.1 ± 51.6	**0.001**	0.277	0.253
*ucp1*	6.41 ± 0.78	4.37 ± 1.03	2.76 ± 1.19	2.95 ± 0.91	**0.024**	0.375	0.288
*pcna*	5.63 ± 0.39	5.23 ± 0.30	5.08 ± 0.58^*^	2.64 ± 0.46	**0.003**	**0.006**	**0.038**
*ocln*	4.73 ± 0.63	4.34 ± 0.48	9.08 ± 1.73	11.4 ± 1.33	**<0.001**	0.409	0.247
*cldn15*	29.0 ± 3.70	26.0 ± 2.72	54.8 ± 10.8	60.7 ± 9.81	**<0.001**	0.843	0.562
*cdh1*	16.2 ± 1.52^*^	12.5 ± 0.99	12.1 ± 2.22	15.0 ± 1.41	0.606	0.786	**0.043**
*cdh17*	57.5 ± 6.76^*^	39.4 ± 3.16	28.1 ± 9.55	27.8 ± 1.68	**0.001**	0.124	0.136
*alpi*	119.6 ± 13.2	106.3 ± 17.5	19.4 ± 6.24	23.4 ± 1.89	**<0.001**	0.699	0.473
*fabp1*	67.4 ± 5.48	75.0 ± 5.98	20.2 ± 12.8	19.5 ± 6.25	**<0.001**	0.674	0.609
*fabp2*	306.4 ± 49.2^*^	159.2 ± 32.7	83.8 ± 47.4	97.6 ± 29.0	**0.001**	0.103	0.051
*fabp6*	–	–	2491.5 ± 995.1	1744.5 ± 331.7	**<0.001**	0.460	0.460
*muc2*	39.1 ± 4.97^*^	26.8 ± 2.66	39.6 ± 12.8	32.3 ± 3.23	0.661	0.162	0.716
*muc13*	82.6 ± 7.89	95.0 ± 24.3	92.5 ± 27.4	76.6 ± 5.89	0.822	0.925	0.460
*hes1-b*	2.94 ± 0.34^*^	1.88 ± 0.17	3.11 ± 0.61	3.93 ± 0.64	**0.029**	0.802	0.061
*tnfα*	0.12 ± 0.02	0.08 ± 0.01	0.18 ± 0.05	0.16 ± 0.02	**0.014**	0.355	0.619
*il1β*	0.03 ± 0.01^*^	0.02 ± 0.0	0.05 ± 0.01	0.06 ± 0.01	**<0.001**	0.672	0.070
*il6*	0.01 ± 0.0	0.02 ± 0.0	0.02 ± 0.01	0.02 ± 0.00	0.134	0.486	0.725
*il8*	0.21 ± 0.03	0.22 ± 0.05	0.37 ± 0.09	0.31 ± 0.03	**0.046**	0.679	0.563
*il10*	0.12 ± 0.01	0.11 ± 0.01	0.36 ± 0.12	0.22 ± 0.03	**0.004**	0.192	0.242
*cd4*	0.26 ± 0.04	0.22 ± 0.03	0.71 ± 0.15	0.60 ± 0.05	**<0.001**	0.363	0.653
*cd8a*	0.54 ± 0.09	0.36 ± 0.04	0.95 ± 0.23	0.79 ± 0.13	**0.004**	0.233	0.956
*cd8b*	0.07 ± 0.01^*^	0.04 ± 0.01	0.13 ± 0.04	0.11 ± 0.03	**0.006**	0.275	0.915
*lgals1*	5.68 ± 0.44^***^	2.49 ± 0.49	12.0 ± 2.44	12.8 ± 1.64	**<0.001**	0.428	0.210
*lgals8*	3.97 ± 0.34^**^	2.81 ± 0.21	5.70 ± 0.95	6.38 ± 0.81	**<0.001**	0.721	0.168
*sIgM*	1.71 ± 0.67	1.60 ± 0.53	3.69 ± 1.53	3.27 ± 0.84	0.069	0.786	0.872
*sIgT*	0.04 ± 0.01^*^	0.02 ± 0.0	0.07 ± 0.03^*^	0.01 ± 0.0	0.335	**0.004**	0.152
*mIgM*	0.13 ± 0.03	0.11 ± 0.02	0.52 ± 0.13	0.37 ± 0.05	**<0.001**	0.235	0.412
*mIgT*	0.20 ± 0.02^*^	0.12 ± 0.03	0.90 ± 0.20^*^	0.32 ± 0.05	**<0.001**	**0.002**	**0.013**
*sIgM/mIgT*	10.2 ± 3.71	12.9 ± 2.89	3.51 ± 1.93	8.32 ± 2.18	**0.050**	0.186	0.706

Values are the mean ± SEM of 6–9 fish. P-values are the result of two-way analysis of variance. Bold values indicate statistically significant differences P < 0.05. The asterisks indicate statistically significant differences (

* *P < 0.05*,

** *P < 0.01*,

*** *P < 0.001) between strains in a given intestine segment. All data values for each tissue were in reference to the expression level of sirt1 of strain 1 in the anterior intestine segment with an arbitrary assigned value of 1. Colors correspond to different gene categories defined in Table 1*.

## Discussion

It is now recognized that SIRTs protect cells from ROS-induced damage across a wide range of biological systems, although the fine regulation of their expression and activity in maintaining cellular homeostasis is not fully understood (Santos et al., 2016). The ultimate mechanisms driving these processes at the cellular level were not addressed in this fish study. However, the integration of transcriptomic profiles from two genetically different gilthead sea bream strains with differences in growth performance contributes to linking the molecular signature of SIRTs to downstream markers of energy and lipid metabolism and immunological/inflammatory status, which allows for improvement in intestinal health and more efficient nutrient utilization. Certainly, in our experimental model, the highest feed intake and FE of fish from strain 1 was related to changes in blood-biochemical indicators of nutritional condition, such as total plasma protein and cholesterol levels, as previously reported for this (Sala-Rabanal et al., 2003; Peres et al., 2013) and other fish species (Congleton and Wagner, 2006; Chatzifotis et al., 2010). The same relationship held true for markers of the GH/IGF axis, and we found that plasma level of IGF-I closely reflected differences in growth potentiality between fish strains, as previously observed when comparing the growth performance of gilthead sea bream with that of the stress sensitive common dentex (Bermejo-Nogales et al., 2007). Experimental evidence regarding the gilthead sea bream also indicates that IGF-I is highly responsive to changes in growth performance due to biotic and abiotic factors, including season and developmental stage (Mingarro et al., 2002; Saera-Vila et al., 2007), ration size (Pérez-Sánchez et al., 1995), crowding and handling stress (Rotllant et al., 2001), physical activity (Vélez et al., 2016), hypoxia (Martos-Sitcha et al., 2017) and dietary protein and lipid source (Gómez-Requeni et al., 2004; Benedito-Palos et al., 2007; Ballester-Lozano et al., 2015; Simó-Mirabet et al., 2017b). Most of these changes in circulating levels of IGF-I are inversely correlated with plasma levels of GH due to the IGF-I feedback inhibition of pituitary GH synthesis and secretion (Pérez-Sánchez, 2000). The same trend was observed herein, although it was not statistically significant. However, it is noteworthy that the trend occurred along with changes in organosomatic indexes, which suggests an enhanced flux of lipids from mesenteric adipose tissue toward the liver and perhaps skeletal muscle. This flux would be mediated, at least in part, by the lipolytic action of GH, which protects tissues from excessive lipid deposition when energy is largely available (Pérez-Sánchez, 2000).

The liver is a key metabolic organ with a remarkable capacity for regeneration based on the assumption that hepatocytes sense changes in metabolic loads and react to buffer them, counteracting, for instance, the risk of hepatic steatosis (Hohmann et al., 2014). Certainly, in our experimental model, different anti-steatosic mechanisms could be triggered with the increase in HSI and feed intake in the fast-growing fish strain. First, the FA elongase ELOVL5 is known to control hepatic triglyceride (TG) storage in higher vertebrates, and a modest increase in hepatic ELOVL5 activity in obese mice dramatically reduced hepatic TGs (Tripathy et al., 2014), whereas knockouts of this gene promoted fatty livers (Moon et al., 2009). In obese mice, the effects of this enzyme on TG metabolism are linked to the increased activity of adipocyte TG lipase without affecting FA ß-oxidation (Tripathy et al., 2014). This metabolic situation is similar to that emerging from our gene expression profiling in livers of fish from strain 1, with increased *elovl5* and *atgl* expression in combination with low expression of *cpt1a*, a key step in the mitochondrial uptake of FAs for ß-oxidation. Concurrently, these fish showed a reduced expression of the lipogenic transcription factor *ppar*γ (Schadinger et al., 2005). Because lipogenesis is considered the most energy-demanding process in liver tissue (Rui, 2014), the probable inhibition of this metabolic pathway was also substantiated by a reduced expression of i) master regulators of mitochondrial biogenesis and activity (*pgc1*α), ii) key enzymes (*cs*) of the TCA cycle and iii) enzyme subunits of Complexes I (*nd2*) and IV (*cox1*) of the mitochondrial respiratory chain. In previous gilthead sea bream studies, *pgc1*α has been targeted as a gene showing a high hepatic response to thermal and husbandry stressors (Bermejo-Nogales et al., 2014). Moreover, the down-regulation of *pgc1*α during the fasting inhibition of hepatic lipogenesis has been related to a marked down-regulation of nearly all the components of the OXPHOS pathway (Bermejo-Nogales et al., 2015), several FA elongases (*elovl4, elovl5, elovl6*), and FA desaturases with Δ6 (*fasd2*) and Δ9 (*scd1a* and *scd1b*) activities (Benedito-Palos et al., 2014).

In the present study, we also observed that the expression of hepatic *scd1a* was higher in fish of strain 1, which would prevent the lipotoxic effect of saturated FAs by favoring their conversion to more safely stored mono-unsaturated FAs (Li et al., 2009; Silbernagel et al., 2012). Therefore, at the liver tissue level, different adaptive mechanisms might act in concert to mitigate the detrimental metabolic effects of enhanced feed intake and tissue lipid storage. How this mechanisms are coupled to SIRT regulation remains unclear; however, the co-regulated down-regulation of *sirt1* and *pgc1*α in the liver tissue of fish of strain 1 is noteworthy, as it could be indicative of a reduced energy demand, oxidative metabolism and oxidative stress, as widely demonstrated in rodents (Gerhart-Hines et al., 2007; Austin and St-Pierre, 2012; Santos et al., 2016). The hepatic SIRT profile in response to the enhanced growth of fish of strain 1 (low *sirt1* expression with no changes in the expression of the other *sirt*s) was clearly opposite to that found during short-term fasting (no changes in *sirt1* expression in combination with an overall down-regulation of *sirt2* to *sirt6*) (Simó-Mirabet et al., 2017a). This effect might reflect the complementarity rather than the redundancy of SIRT actions when organisms are facing different types of increased energy demand (i.e., fasting vs. fast growth).

Adipose tissue plays a central role in regulating whole body lipid and energy homeostasis, and it undergoes continuous lipid trafficking to different metabolically active tissues, mostly liver and muscle (Hodson and Fielding, 2010). In our experimental model, the number of differentially expressed genes at the adipose tissue level was relatively low. However, the low MFI of fish of strain 1 suggests an increased flux of FAs from adipose tissue toward liver and muscle rather than low lipid deposition rates. Certainly, *ATGL* encodes for an intracellular TG lipase that is a key enzyme for both lipid storage and mobilization (Schweiger et al., 2006; Hodson and Fielding, 2010), and its increased expression at the adipose tissue level is typical of a lean phenotype in mice (Shimizu et al., 2015). In the present study, we only found a modest up-regulation of *atgl* in the adipose tissue of fish of strain 1, but this up-regulation occurred in association with the up-regulation of *sirt6*. Fat-specific *Sirt6* KO mice promoted high-fat-diet induced obesity by impairing ATGL expression inhibiting the lipolytic activity. In addition, adipose SIRT6 level is decreased in obese human patients (Kuang et al., 2017). Our fast-growing fish also exhibited other lipolytic features such as a high *ppar*α expression, which prevents obesity in mice (Guerre-Millo et al., 2000) and chickens (Ji et al., 2014). Then, the lipolytic state of fish of strain 1 could be mainly orchestrated by the up-regulation of *sirt5* and *sirt6*, whereas short-term fasting up-regulated *sirt1* and down-regulated *sirt2* and *sirt7* (Simó-Mirabet et al., 2017a). Because most lipolytic factors, including PPARα (Delerive et al., 2001; Wahli and Michalik, 2012) and SIRT5-6 (Kuang et al., 2017; Wang et al., 2017) have anti-inflammatory effects, the lean phenotype is largely recognized as a healthy condition in a wide range of animals. Certainly, measures of lean fish based on gross measurements of body fat are currently used in breeding selection programs to produce more efficient fish (Kause et al., 2016); we consider that such approaches can be refined and improved by the gene expression profiling of SIRTs and other metabolic biomarkers of adipose tissue.

White skeletal muscle accounts for up to 60% of the body weight of fish (Johnston et al., 2011) and is a high energy consumer during growth. In the present study, the differences observed in gene expression pattern of white skeletal muscle between strains suggest that the fast-growing strain was metabolically more active and efficient than fish of strain 2. Notably, the muscle of fast-growing fish exhibited high expression levels of *pgc1*α, a well-recognized marker of increased mitochondrial activity and thereby aerobic oxidative capacity (Austin and St-Pierre, 2012; Wenz, 2013). This finding is in contrast to the observations of Robledo et al. (2017) in turbot indicating the up-regulation of the glycolytic pathway in the muscle of selected fast-growing fish, which could reflect changes in energy demand as well as in swimming and feeding behavior. While the increased *pgc1*α expression in fast-growing fish did not occur along with changes in OXPHOS gene expression, it is known that PGC1α-mediated enhance of oxidative capacity may result from an increase in the number of mitochondria (Srivastava et al., 2009) or from the effects of PGC1α on the activity of the enzymes (Austin and St-Pierre, 2012) without altering OXPHOS gene expression. In accordance with the suggested increased oxidative capacity of strain 1, we observed indication of enhanced FA oxidation, such as higher expression of genes coding for enzymes (*lpl* and *atgl*) and transcription factors (*ppar*α) involved in lipoprotein metabolism, tissue FA uptake and TG catabolism. The up-regulation of this lipolytic machinery is a well-known process in both gilthead sea bream and European sea bass during fasting (Benedito-Palos et al., 2014; Rimoldi et al., 2016), which supports the notion that both fasting and enhanced growth are highly demanding energy processes for skeletal muscle. Moreover, the better performance of fish of strain 1 was associated to a down-regulation of the muscle-specific uncoupling protein 3 (*ucp3*). Both in fish and other vertebrates, nutrient and energy overflow activates UCP for protecting mitochondria against oxidative stress (Bermejo-Nogales et al., 2011). Our results may indicate higher metabolic efficiency through a more coupled respiration in this strain, and agree with a higher oxidative capacity. Improved oxidative capacity in higher vertebrates (e.g., through endurance training) down-regulates UCP3 (Schrauwen-Hinderling et al., 2003). Experimental evidence in humans and rodents indicates that SIRT2 integrates changes in energy demand, lipid oxidation and redox homeostasis by increasing FAs oxidation via activation of PGC1α (Krishnan et al., 2012) and activating ROS-scavenging enzymes (Austin and St-Pierre, 2012). Physiological studies in humans also reveal a regulatory role of SIRT2 in muscle stem cell proliferation and differentiation (Dryden et al., 2003; Wu et al., 2014; Stanton et al., 2017). Single nucleotide polymorphism of SIRT2 has also been associated with different body size traits in Quinchuan cattle (Gui et al., 2015). Importantly, we herein found that the expression of muscle *sirt2* was markedly up-regulated in the fast-growing fish strain, whereas it remains mostly unaltered during short-term fasting (Simó-Mirabet et al., 2017a). All of these findings provide further evidence of a differential regulation of cell energy sensors depending on the intensity and type of the energy-demanding stimuli.

The intestinal tract is involved not only in digestion and feed absorption but also in water and electrolyte balance, nutrient sensing and immunity (Cain and Swan, 2010). This diversity is now starting to be elucidated, and microarray gene expression profiling of European sea bass intestine revealed pronounced spatial transcriptional changes with an over-representation of nutrient transporters and mucosal chemosensors of intestinal motility and secretion in anterior-medium intestine segments, whereas immunity markers are highly over-expressed in the posterior intestine segment (Calduch-Giner et al., 2016). This expression pattern has also been inferred for gilthead sea bream in both this and previous studies (Pérez-Sánchez et al., 2015; Estensoro et al., 2016; Simó-Mirabet et al., 2017b) using intestinal PCR-arrays of selected markers of intestinal architecture and function. Moreover, the expression pattern of the fast-growing strain appears to be better suited to cope with enhanced feed intake and growth rates, as inferred by the up-regulated expression in the anterior intestine segment of genes involved in cell adhesion and epithelial integrity (*cdh1* and *cdh17*), mucus production (*muc2*), Goblet cell differentiation (*hes1-b*) and FA transport (*fabp2*). Intriguingly, this molecular feature is concurrent with the down-regulation of *sirt4*. Unlike other SIRT family members, SIRT4 exhibits no deacetylation activity, and this novel regulator of lipid homeostasis is active in nutrient-replete conditions for repressing FA oxidation while activating lipogenesis (Laurent et al., 2013). Accordingly, SIRT4 knockdown leads to increased FA oxidation in liver and muscle tissues (Nasrin et al., 2010), and low circulating levels of SIRT4 mirror attempts to increase FA oxidation in obese humans (Tarantino et al., 2014). To our knowledge, few studies have addressed the regulation of SIRT4 at the intestine level. However, the intestinal down-regulation of *sirt4* in fast-growing fish with enhanced feed intake might indicate a protective mechanism for avoiding the damaging effects of excessive accumulation of lipid droplets in enterocytes, which would be counter-regulated by the reduced expression of *cpt1a*, a key limiting enzyme of mitochondrial FA uptake and β-oxidation. Together, these results highlight the potential use of intestinal SIRT4 as a biomarker of diagnostic as well as predictor of growth potentiality and nutritional condition, particularly when used in combination with other nutritionally regulated biomarkers of blood biochemistry and tissue histo-pathological data scoring (Ballester-Lozano et al., 2015).

Regarding markers of cell proliferation and immunity, we also observed different gene expression patterns across the intestine of fish with differences in growth performance. A significant increase in the expression of both secreted and membrane *IgT* was observed in fast-growing fish. *IgT* is the key mucosal immunoglobulin in teleost fish (Zhang et al., 2010), and the importance of its fine regulation upon infection has been recently described in fish with different nutritional backgrounds (Piazzon et al., 2016). There is also evidence of enhanced expression of *IgT* in the intestine of fish fed with the probiotic *Bacillus amyloliquefaciens* CET 5940 (Simó-Mirabet et al., 2017b), leading to better disease outcomes in fish challenged with the intestinal parasite *Enteromyxum leei* (Piazzon et al., 2016). Because anti-inflammatory action has been reported in rodents and humans for most SIRT isotypes, including SIRT2 (Wang et al., 2016), SIRT3 (Liu et al., 2012, 2015), SIRT5 (Tannahill, 2013; Qin et al., 2017) and SIRT7 (Vakhrusheva et al., 2008), their enhanced expression in the posterior intestine of gilthead sea bream can be considered a preventive response to keep regulated the immune system of fish with a pre-stimulatory condition, as determined by the enhanced expression of markers of OXPHOS pathway (*nd2*) and cell proliferation (*pcna*). Likewise, in the intestine of gilthead sea bream, the anti-inflammatory action of the *Bacillus* probiotic was related to an overall decrease in SIRT gene expression for *sirt1, sirt2, sirt3*, and *sirt7* (Simó-Mirabet et al., 2017b).

**Figure 3:**
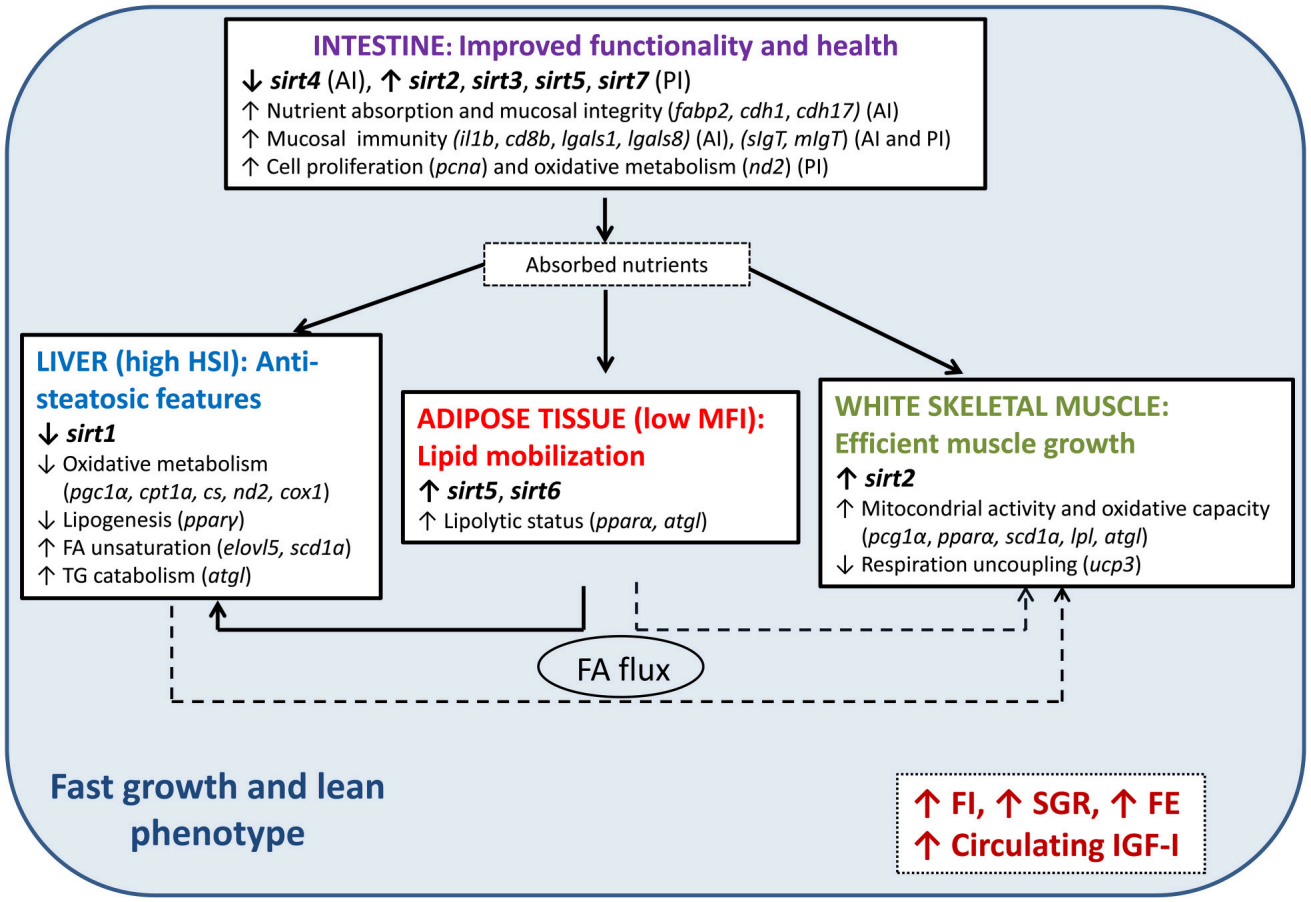
Tissue-specific expression patterns of SIRTs and the inferred metabolic features leading to a fast-growing and lean phenotype in gilthead sea bream. Arrows indicate the direction of change in metabolic processes and expression of the indicated genes. Only the genes that are the most informative about these metabolic features are shown in parentheses. HSI, hepatosomatic index; MFI, mesenteric fat index; FI, feed intake; SGR, specific growth rate; FE, feed efficiency; IGF-I, plasma insulin growth factor-1; FA, fatty acid; AI, anterior intestine; PI, posterior intestine; TG, triglyceride. For gene names, refer to Table 1.

In summary, as shown in Figure 3, this study illustrates the metabolic crosstalk among different tissues, identifying metabolic features that led to a lean, fast-growing and feed-efficient fish phenotype. These metabolic features are accompanied by tissue-specific mechanisms that would protect the organisms against possible lipotoxicity, oxidative stress and inflammation, processes that are related to a particular tissue-specific SIRTs expression pattern. Accordingly, in our model of fast-growing fish, several SIRT isotypes may play anti-inflammatory roles in the intestine (*sirt2, 3, 5*, and *7*) and adipose tissue (*sirt5* and *6*), also favoring an increased flux of lipids from adipose tissue toward the liver and perhaps skeletal muscle. At the same time, high expression levels of s*irt2* may play a role in accelerated muscle growth in combination with an enhanced FA oxidative capacity and reduced hepatic lipogenesis, which might be sensed by reduced hepatic *sirt1* expression. As is typical in terrestrial livestock animals, lean farmed fish appear to be highly efficient and visceral fat content is currently used for indirect selection of improved feed conversion ratio in salmonids (Kause et al., 2016). The advantage of using SIRTs and SIRT-related biomarkers has been discussed to improve and refine the genetic selection programs of farmed fish to finely discriminate among low fat measurements that may arise from reduced feed intake, nutritional imbalances or any other metabolic dysfunction. However, further research is still needed to clarify whether differences in the SIRT profile between fish strains results from genetic or epigenetic sources of variation affecting the regulation of SIRTs at the transcriptional or protein level, or from the action of other genes leading to different pathways upstream of SIRTs.

## Author contributions

JP-S conceived and designed the study. PS-M, JC-G, and JA performed the experiments. PS-M, EP, JC-G, JA, and JP-S analyzed the data. PS-M, EP, and JP-S wrote the manuscript.

### Conflict of interest statement

The authors declare that the research was conducted in the absence of any commercial or financial relationships that could be construed as a potential conflict of interest.

## References

[B1] Austin S. St-Pierre J. (2012). PGC1α and mitochondrial metabolism–emerging concepts and relevance in ageing and neurodegenerative disorders. J. Cell. Sci. 125, 4963–4971. 10.1242/jcs.113662 23277535

[B2] Ballester-Lozano G. F. Benedito-Palos L. Estensoro I. Sitjà-Bobadilla A. Kaushik S. Pérez-Sánchez J. (2015). Comprehensive biometric, biochemical and histopathological assessment of nutrient deficiencies in gilthead sea bream fed semi-purified diets. Br. J. Nutr. 114, 713–726. 10.1017/S0007114515002354 26220446

[B3] Benedito-Palos L. Ballester-Lozano G. Pérez-Sánchez J. (2014). Wide gene expression analysis of lipid-relevant genes in nutritionally challenged gilthead sea bream (*Sparus aurata*). Gene 547, 34–42. 10.1016/j.gene.2014.05.073 24946022

[B4] Benedito-Palos L. Saera-Vila A. Calduch-Giner J. Sadasivam K. Pérez-Sánchez J. (2007). Combined replacement of fish meal and oil in practical diets for fast growing juveniles of gilthead sea bream (*Sparus aurata* L.): networking of systemic and local components of GH/IGF axis. Aquaculture 267, 199–212. 10.1016/j.aquaculture.2007.01.011

[B5] Bermejo-Nogales A. Benedito-Palos L. Calduch-Giner J. A. Pérez-Sánchez J. (2011). Feed restriction up-regulates uncoupling protein 3 (UCP3) gene expression in heart and red muscle tissues of gilthead sea bream (*Sparus aurata* L.): new insights in substrate oxidation and energy expenditure. Comp. Biochem. Phys. A 159, 296–302. 10.1016/j.cbpa.2011.03.024 21463702

[B6] Bermejo-Nogales A. Calduch-Giner J. A. Pérez-Sánchez J. (2015). Unraveling the molecular signatures of oxidative phosphorylation to cope with the nutritionally changing metabolic capabilities of liver and muscle tissues in farmed fish. PLoS ONE 10:e0122889. 10.1371/journal.pone.0122889 25875231 PMC4398389

[B7] Bermejo-Nogales A. Nederlof M. Benedito-Palos L. Ballester-Lozano G. F. Folkedal O. Olsen R. E.. (2014). Metabolic and transcriptional responses of gilthead sea bream (*Sparus aurata* L.) to environmental stress: new insights in fish mitochondrial phenotyping. Gen. Comp. Endocrinol. 205, 305–315. 10.1016/j.ygcen.2014.04.016 24792819

[B8] Bermejo-Nogales A. Saera-Vila A. Calduch-Giner J. A. Navarro J. C. Sitjà-Bobadilla A. Pérez-Sánchez J. (2007). Differential metabolic and gene expression profile of juvenile common dentex (*Dentex dentex* L.) and gilthead sea bream (*Sparus aurata* L.) in relation to redox homeostasis. Aquaculture 267, 213–224. 10.1016/j.aquaculture.2007.01.024

[B9] Cain K. Swan C. (2010). Barrier function and immunology, in Fish Physiology, Vol. 30, eds Grosell M. Farrell A. P. Brauner C. J. (San Diego, CA: Academic Press), 111–134.

[B10] Calduch-Giner J. A. Sitjà-Bobadilla A. Pérez-Sánchez J. (2016). Gene expression profiling reveals functional specialization along the intestinal tract of a carnivorous teleostean fish (*Dicentrarchus labrax*). Front. Physiol. 7:359. 10.3389/fphys.2016.00359 27610085 PMC4997091

[B11] Cardoso D. C. Martinati J. C. Giachetto P. F. Vidal R. O. Carazzolle M. F. Padilha L.. (2014). Large-scale analysis of differential gene expression in coffee genotypes resistant and susceptible to leaf miner-toward the identification of candidate genes for marker assisted-selection. BMC Genomics 15:66. 10.1186/1471-2164-15-66 24460833 PMC3924705

[B12] Chatzifotis S. Panagiotidou M. Papaioannou N. Pavlidis M. Nengas I. Mylonas C. C. (2010). Effect of dietary lipid levels on growth, feed utilization, body composition and serum metabolites of meagre (*Argyrosomus regius*) juveniles. Aquaculture 307, 65–70. 10.1016/j.aquaculture.2010.07.002

[B13] Chen Y. Gondro C. Quinn K. Herd R. M. Parnell P. F. Vanselow B. (2011). Global gene expression profiling reveals genes expressed differentially in cattle with high and low residual feed intake. Anim. Genet. 42, 475–490. 10.1111/j.1365-2052.2011.02182.x 21906099

[B14] Choi M.-J. Kim G.-D. Kim J.-M. Lim H. K. (2015). Differentially-expressed genes associated with faster growth of the Pacific Abalone, *Haliotis discus* hannai. Int. J. Mol. Sci. 16, 27520–27534. 10.3390/ijms161126042 26593905 PMC4661900

[B15] Choudhary C. Weinert B. T. Nishida Y. Verdin E. Mann M. (2014). The growing landscape of lysine acetylation links metabolism and cell signalling. Nat. Rev. Mol. Cell Biol. 15, 536–550. 10.1038/nrm3841 25053359

[B16] Congleton J. L. Wagner T. (2006). Blood-chemistry indicators of nutritional status in juvenile salmonids. J. Fish Biol. 69, 473–490. 10.1111/j.1095-8649.2006.01114.x

[B17] Connon R. E. Deanovic L. A. Fritsch E. B. D'Abronzo L. S. Werner I. (2011). Sublethal responses to ammonia exposure in the endangered delta smelt; *Hypomesus transpacificus* (Fam. Osmeridae). Aquat. Toxicol. 105, 369–377. 10.1016/j.aquatox.2011.07.002 21820383

[B18] Danzmann R. G. Kocmarek A. L. Norman J. D. Rexroad C. E. Palti Y. (2016). Transcriptome profiling in fast versus slow-growing rainbow trout across seasonal gradients. BMC Genomics 17:60. 10.1186/s12864-016-2363-5 26768650 PMC4714434

[B19] Delerive P. Fruchart J. C. Staels B. (2001). Peroxisome proliferator-activated receptors in inflammation control. Int. J. Endocrinol. 169, 453–459. 10.1677/joe.0.1690453 11375115

[B20] Dryden S. C. Nahhas F. A. Nowak J. E. Goustin A. S. Tainsky M. A. (2003). Role for human SIRT2 NAD-dependent deacetylase activity in control of mitotic exit in the cell cycle. Mol. Cell. Biol. 23, 3173–3185. 10.1128/MCB.23.9.3173-3185.2003 12697818 PMC153197

[B21] Ekambaram P. Parasuraman P. (2017). Differential expression of sirtuin 2 and adipocyte maturation restriction: an adaptation process during hypoxia in fish. Biol. Open 6, 1375–1382. 10.1242/bio.027334 28808139 PMC5612243

[B22] Estensoro I. Ballester-Lozano G. Benedito-Palos L. Grammes F. Martos-Sitcha J. A. Mydland L.-T.. (2016). Dietary butyrate helps to restore the intestinal status of a marine teleost (*Sparus aurata*) fed extreme diets low in fish meal and fish oil. PLoS ONE 11:e0166564. 10.1371/journal.pone.0166564 27898676 PMC5127657

[B23] Fernández-Díaz C. Kopecka J. Cañavate J. P. Sarasquete C. Solé M. (2006). Variations on development and stress defences in *Solea senegalensis* larvae fed on live and microencapsulated diets. Aquaculture 251, 573–584. 10.1016/j.aquaculture.2005.06.014

[B24] Frye R. A. (2000). Phylogenetic classification of prokaryotic and eukaryotic Sir2-like proteins. Biochem. Biophys. Res. Commun. 273, 793–798. 10.1006/bbrc.2000.3000 10873683

[B25] Gerhart-Hines Z. Rodgers J. Bare O. Lerin C. Kim S.-H. Mostoslavsky R. (2007). Metabolic control of muscle mitochondrial function and fatty acid oxidation through SIRT1/PGC-1 alpha. EMBO J. 26, 1913–1923. 10.1038/sj.emboj.7601633 17347648 PMC1847661

[B26] Ghinis-Hozumi Y. Antaramian A. Villarroya F. Piña E. Mora O. (2013). Potential role of sirtuins in livestock production. Animal 7, 101–108. 10.1017/S1751731112001115 23031219

[B27] Ghosh H. S. McBurney M. Robbins P. D. (2010). SIRT1 negatively regulates the mammalian target of rapamycin. PLoS ONE 5:e9199. 10.1371/journal.pone.0009199 20169165 PMC2821410

[B28] Gjedrem T. Baranski M. (2009). Selective Breeding in Aquaculture: An Introduction. Dordrecht: Springer Science & Business Media.

[B29] Gomes P. Outeiro T. F. Cavadas C. (2015). Emerging role of Sirtuin 2 in the regulation of mammalian metabolism. Trends Pharmacol. Sci. 36, 756–768. 10.1016/j.tips.2015.08.001 26538315

[B30] Gómez-Requeni P. Mingarro M. Calduch-Giner J. A. Médale F. Martin S. A. M. Houlihan D. F. (2004). Protein growth performance, amino acid utilization and somatotropic axis responsiveness to fishmeal replacement by plant protein sources in gilthead sea bream (*Sparus aurata*). Aquaculture 232, 493–510. 10.1016/S0044-8486(03)00532-5

[B31] Greiss S. Gartner A. (2009). Sirtuin/Sir2 phylogeny, evolutionary considerations and structural conservation. Mol. Cells 28, 407–415. 10.1007/s10059-009-0169-x 19936627 PMC3710699

[B32] Guan K. L. Xiong Y. (2011). Regulation of intermediary metabolism by protein acetylation. Trends Biochem. Sci. 36, 108–116. 10.1016/j.tibs.2010.09.003 20934340 PMC3038179

[B33] Guerre-Millo M. Gervois P. Raspe E. Madsen L. Poulain P. Derudas B.. (2000). Peroxisome proliferator-activated receptor α activators improve insulin sensitivity and reduce adiposity. J. Biol. Chem. 275, 16638–16642. 10.1074/jbc.275.22.16638 10828060

[B34] Gui L. Hao R. Zhang Y. Zhao X. Zan L. (2015). Haplotype distribution in the class I sirtuin genes and their associations with ultrasound carcass traits in Qinchuan cattle (*Bos taurus*). Molec. Cell. Probes 29, 102–107. 10.1016/j.mcp.2015.03.007 25839883

[B35] Hodson L. Fielding B. A. (2010). Trafficking and partitioning of fatty acids: the transition from fasted to fed state. Clin. Lipidol. 5, 131–144. 10.2217/clp.09.72

[B36] Hohmann N. Weiwei W. Dahmen U. Dirsch O. Deutsch A. Voss-Böhme A. (2014). How does a single cell know when the liver has reached its correct size? PLoS ONE 9:e93207. 10.1371/journal.pone.0093207 24690888 PMC3972176

[B37] Houtkooper R. H. Pirinen E. Auwerx J. (2012). Sirtuins as regulators of metabolism and healthspan. Nat. Rev. Mol. Cell. Biol. 13, 225–238. 10.1038/nrm3293 22395773 PMC4872805

[B38] Janssen K. Chavanne H. Berentsen P. Komen H. (2015). Gilthead Seabream (Sparus aurata)–Current status of selective breeding in Europe. Available online at: http://www.fishboost.eu/reports-on-current-status-of-selective-breeding-in-europe.html (Accessed on January 2018)

[B39] Ji B. Middleton J. L. Ernest B. Saxton A. M. Lamont S. J. Campagna S. R. (2014). Molecular and metabolic profiles suggest that increased lipid catabolism in adipose tissue contributes to leanness in domestic chicken. Physiol. Genomics 46, 315–327. 10.1152/physiolgenomics.00163.2013 24550212

[B40] Jing H. Lin H. (2015). Sirtuins in epigenetic regulation. Chem. Rev. 115, 2350–2375. 10.1021/cr500457h 25804908 PMC4610301

[B41] Johnston I. A. Bower N. I. Macqueen D. J. (2011). Growth and the regulation of myotomal muscle mass in teleost fish. J. Exp. Biol. 214, 1617–1628. 10.1242/jeb.038620 21525308

[B42] Kause A. Kiessling A. Martin S. A. M. Houlihan D. Ruohonen K. (2016). Genetic improvement of feed conversion ratio via indirect selection against lipid deposition in farmed rainbow trout (*Oncorhynchus mykiss* Walbaum). Br. J. Nutr. 116, 1656–1665. 10.1017/S0007114516003603 27813470

[B43] Krishnan J. Danzer C. Simka T. Ukropec J. Walter K. M. Kumpf S. (2012). Dietary obesity-associated Hif1a activation in adipocytes restricts fatty acid oxidation and energy expenditure via suppression of the Sirt2-NAD+ system. Genes Dev. 26, 259–270. 10.1101/gad.180406.111 22302938 PMC3278893

[B44] Kuang J. Zhang Y. Liu Q. Shen J. Pu S. Cheng S.. (2017). Fat-specific Sirt6 ablation sensitizes mice to high-fat diet-induced obesity and insulin resistance by inhibiting lipolysis. Diabetes 66, 1159–1171. 10.2337/db16-1225 28250020

[B45] Laplante M. Sabatini D. M. (2012). mTOR signaling in growth control and disease. Cell 149, 274–293. 10.1016/j.cell.2012.03.017 22500797 PMC3331679

[B46] Laurent G. de Boer V. C. Finley L. W. Sweeney M. Lu H. Schug T. T.. (2013). SIRT4 represses peroxisome proliferator-activated receptor α activity to suppress hepatic fat oxidation. Mol. Cell. Biol. 33, 4552–4561. 10.1128/MCB.00087-13 24043310 PMC3838178

[B47] Lee-Montero I. Navarro A. Borrell Y. García-Celdrán M. Martín N. Negrín-Báez D.. (2013). Development of the first standardised panel of two new microsatellite multiplex PCRs for gilthead seabream (*Sparus aurata* L.). Anim. Genet. 44, 533–546. 10.1111/age.12037 23574152

[B48] Li Z. Z. Berk M. McIntyre T. M. Feldstein A. E. (2009). Hepatic lipid partitioning and liver damage in nonalcoholic fatty liver disease: role of stearoyl-CoA desaturase. J. Biol. Chem. 284, 5637–5644. 10.1074/jbc.M807616200 19119140 PMC2645822

[B49] Liu T. F. Brown C. M. El Gazzar M. McPhail L. Millet P. Rao A.. (2012). Fueling the flame: bioenergy couples metabolism and inflammation. J. Leukoc. Biol. 92, 499–507. 10.1189/jlb.0212078 22571857 PMC3427613

[B50] Liu T. F. Vachharajani V. Millet P. Bharadwaj M. S. Molina A. J. McCall C. E. (2015). Sequential actions of SIRT1-RELB-SIRT3 coordinate nuclear-mitochondrial communication during immunometabolic adaptation to acute inflammation and sepsis. J. Biol. Chem. 290, 396–408. 10.1074/jbc.M114.566349 25404738 PMC4281742

[B51] Livak K. J. Schmittgen T. D. (2001). Analysis of relative gene expression data using real-time quantitative PCR and the 2-ΔΔCT. Methods 25, 402–408. 10.1006/meth.2001.1262 11846609

[B52] Lundby A. Lage K. Weinert B. T. Bekker-Jensen D. B. Secher A. Skovgaard T.. (2012). Proteomic analysis of lysine acetylation sites in rat tissues reveals organ specificity and subcellular patterns. Cell Rep. 2, 419–431. 10.1016/j.celrep.2012.07.006 22902405 PMC4103158

[B53] Mailloux R. J. Harper M. E. (2011). Uncoupling proteins and the control of mitochondrial reactive oxygen species production. Free Radic. Biol. Med. 51, 1106–1115. 10.1016/j.freeradbiomed.2011.06.022 21762777

[B54] Martínez-Álvarez R. M. Morales A. E. Sanz A. (2005). Antioxidant defenses in fish: biotic and abiotic factors. Rev. Fish Biol. Fish. 15, 75–88. 10.1007/s11160-005-7846-4

[B55] Martínez-Barberá J. P. Pendón C. Martí-Palanca H. Calduch-Giner J. A. Rodríguez R. B. Valdivia M. M.. (1995). The use of recombinant gilthead sea bream (*Sparus aurata*) growth hormone for radioiodination and standard preparation in radioimmunoassay. Comp. Biochem. Physiol. A Physiol. 110, 335–340. 10.1016/0300-9629(94)00178-V 7669108

[B56] Martin S. A. M. Król E. (2017). Nutrigenomics and immune function in fish: new insights from omics technologies. Dev. Comp. Immunol. 75, 86–98. 10.1016/j.dci.2017.02.024 28254621 PMC5495911

[B57] Martos-Sitcha J. A. Bermejo-Nogales A. Calduch-Giner J. A. Pérez-Sánchez J. (2017). Gene expression profiling of whole blood cells supports a more efficient mitochondrial respiration in hypoxia-challenged gilthead sea bream (*Sparus aurata*). Front. Zool. 14:34. 10.1186/s12983-017-0220-2 28694839 PMC5501551

[B58] Masri S. (2015). Sirtuin-dependent clock control: new advances in metabolism, aging and cancer. Curr. Opin. Clin. Nutr. Metab. Care 18, 521–527. 10.1097/MCO.0000000000000219 26335311 PMC4610809

[B59] McAndrew B. Napier J. (2011). Application of genetics and genomics to aquaculture development: current and future directions. J. Agric. Sci. 149, 143–151. 10.1017/S0021859610001152

[B60] Mingarro M. de Celis S. V. R. Astola A. Pendón C. Valdivia M. M. Pérez-Sánchez J. (2002). Endocrine mediators of seasonal growth in gilthead sea bream (*Sparus aurata*): the growth hormone and somatolactin paradigm. Gen. Comp. Endocrinol. 128, 102–111. 10.1016/S0016-6480(02)00042-4 12392683

[B61] Moon Y. A. Hammer R. E. Horton J. D. (2009). Deletion of ELOVL5 leads to fatty liver through activation of SREBP-1c in mice. J. Lipid Res. 50, 412–423. 10.1194/jlr.M800383-JLR200 18838740 PMC2638104

[B62] Nabben M. Hoeks J. (2008). Mitochondrial uncoupling protein 3 and its role in cardiac-and skeletal muscle metabolism. Physiol. Behav. 94, 259–269. 10.1016/j.physbeh.2007.11.039 18191161

[B63] Nasrin N. Wu X. Fortier E. Feng Y. Bare O. C. Chen S.. (2010). SIRT4 regulates fatty acid oxidation and mitochondrial gene expression in liver and muscle cells. J. Biol. Chem. 285, 31995–32002. 10.1074/jbc.M110.124164 20685656 PMC2952200

[B64] Nei M. (1973). Analysis of gene diversity in subdivided populations. Proc. Natl. Acad. Sci. U.S.A. 70, 3321–3323. 10.1073/pnas.70.12.3321 4519626 PMC427228

[B65] Olesen I. Bentsen H. B. Phillips M. Ponzoni R. W. (2015). Can the global adoption of genetically improved farmed fish increase beyond 10%, and how? J. Mar. Sci. Eng. 3, 240–266. 10.3390/jmse3020240

[B66] Otero-Rodiño C. Librán-Pérez M. Velasco C. Álvarez-Otero R. López-Patiño M. A. Míguez J. M.. (2016). Glucosensing in liver and Brockmann bodies of rainbow trout through glucokinase-independent mechanisms. Comp. Biochem. Physiol. B Biochem. Mol. Biol. 199, 29–42. 10.1016/j.cbpb.2015.09.008 26439857

[B67] Pacitti D. Wang T. Martin S. A. M. Sweetman J. Secombes C. J. (2014). Insights into the fish thioredoxin system: expression profile of thioredoxin and thioredoxin reductase in rainbow trout (*Oncorhynchus mykiss*) during infection and *in vitro* stimulation. Dev. Comp. Immunol. 42, 261–277. 10.1016/j.dci.2013.09.013 24095766

[B68] Peres H. Santos S. Oliva-Teles A. (2013). Selected plasma biochemistry parameters in gilthead seabream (*Sparus aurata*) juveniles. J. Appl. Ichthyol. 29, 630–636. 10.1111/j.1439-0426.2012.02049.x

[B69] Pérez-Sánchez J. (2000). The involvement of growth hormone in growth regulation, energy homeostasis and immune function in the gilthead sea bream (*Sparus aurata*): a short review. Fish Physiol. Biochem. 22, 135–144. 10.1023/A:1007816015345

[B70] Pérez-Sánchez J. Benedito-Palos L. Estensoro I. Petropoulos Y. Calduch-Giner J. A. Browdy C. L.. (2015). Effects of dietary NEXT ENHANCE® 150 on growth performance and expression of immune and intestinal integrity related genes in gilthead sea bream (*Sparus aurata* L.). Fish Shellfish Immunol. 44, 117–128. 10.1016/j.fsi.2015.01.039 25681752

[B71] Pérez-Sánchez J. Marti-Palanca H. Kaushik S. J. (1995). Ration size and protein intake affect circulating growth hormone concentration, hepatic growth hormone binding and plasma insulin-like growth factor-I immunoreactivity in a marine teleost, the gilthead sea bream (*Sparus aurata*). J. Nutr. 125, 546–552. 7876930 10.1093/jn/125.3.546

[B72] Piazzon M. C. Calduch-Giner J. A. Fouz B. Estensoro I. Simó-Mirabet P. Puyalto M.. (2017). Under control: how a dietary additive can restore the gut microbiome and proteomic profile, and improve disease resilience in a marine teleostean fish fed vegetable diets. Microbiome 5:164. 10.1186/s40168-017-0390-3 29282153 PMC5745981

[B73] Piazzon M. C. Galindo-Villegas J. Pereiro P. Estensoro I. Calduch-Giner J. A. Gomez-Casado E.. (2016). Differential modulation of IgT and IgM upon parasitic, bacterial, viral, and dietary challenges in a Perciform fish. Front. Immunol. 7:637. 10.3389/fimmu.2016.00637 28082977 PMC5186763

[B74] Qin K. Han C. Zhang H. Li T. Li N. Cao X. (2017). NAD+ dependent deacetylase Sirtuin 5 rescues the innate inflammatory response of endotoxin tolerant macrophages by promoting acetylation of p65. J. Autoimmun. 81, 120–129. 10.1016/j.jaut.2017.04.006 28461090

[B75] Rajan K. E. Thangaleela S. Balasundaram C. (2015). Spatial learning associated with stimulus response in goldfish *Carassius auratus:* relationship to activation of CREB signalling. Fish. Physiol. Biochem. 41, 685–694. 10.1007/s10695-015-0038-9 25739351

[B76] Raymond M. Rousset F. (1995). Genepop (Version 1.2): population-genetics software for exact tests and ecumenicism. J. Hered. 86, 248–249. 10.1093/oxfordjournals.jhered.a111573

[B77] Rimoldi S. Benedito-Palos L. Terova G. Pérez-Sánchez J. (2016). Wide-targeted gene expression infers tissue-specific molecular signatures of lipid metabolism in fed and fasted fish. Rev. Fish Biol. Fish. 26, 93–108. 10.1007/s11160-015-9408-8

[B78] Rise M. L. Hall J. R. Nash G. W. Xue X. Booman M. Katan T.. (2015). Transcriptome profiling reveals that feeding wild zooplankton to larval Atlantic cod (*Gadus morhua*) influences suites of genes involved in oxidation-reduction, mitosis, and selenium homeostasis. BMC Genomics 16:1016. 10.1186/s12864-015-2120-1 26610852 PMC4661974

[B79] Robledo D. Rubiolo J. A. Cabaleiro S. Martínez P. Bouza C. (2017). Differential gene expression and SNP association between fast- and slow-growing turbot (*Scophthalmus maximus*). Sci. Rep. 7:12105. 10.1038/s41598-017-12459-4 28935875 PMC5608734

[B80] Rotllant J. Balm P. H. M. Pérez-Sánchez J. Wendelaar-Bonga S. E. Tort L. (2001). Pituitary and interrenal function in gilthead sea bream (*Sparus aurata* L., Teleostei) after handling and confinement stress. Gen. Comp. Endocrinol. 121, 333–342. 10.1006/gcen.2001.7604 11254375

[B81] Rousset F. (2008). Genepop'007: a complete re-implementation of the genepop software for Windows and Linux. Mol. Ecol. Resour. 8, 103–106. 10.1111/j.1471-8286.2007.01931.x 21585727

[B82] Rui L. (2014). Energy metabolism in the liver. Compr. Physiol. 4, 177–197. 10.1002/cphy.c130024 24692138 PMC4050641

[B83] Saera-Vila A. Calduch-Giner J. Pérez-Sánchez. (2007). Co-expression of IGFs and GH receptors (GHRs) in gilthead sea bream (*Sparus aurata* L.): sequence analysis of the GHR-flanking region. J. Endocrinol. 194, 361–372. 10.1677/JOE-06-0229 17641285

[B84] Sala-Rabanal M. Sánchez J. Ibarz A. Fernández-Borràs J. Blasco J. Gallardo M. A. (2003). Effects of low temperatures and fasting on hematology and plasma composition of gilthead sea bream (*Sparus aurata*). Fish Physiol. Biochem. 29, 105–115. 10.1023/B:FISH.0000035904.16686.b6

[B85] Santos L. Escande C. Denicola A. (2016). Potential modulation of sirtuins by oxidative stress. Oxid. Med. Cell. Longev. 2016:9831825. 10.1155/2016/9831825 26788256 PMC4691645

[B86] Saravanan S. Schrama J. W. Figueiredo-Silva A. C. Kaushik S. J. Verreth J. A. Geurden I. (2012). Constraints on energy intake in fish: the link between diet composition, energy metabolism, and energy intake in rainbow trout. PLoS ONE 7:e34743. 10.1371/journal.pone.0034743 22496852 PMC3322127

[B87] Schadinger S. E. Bucher N. L. Schreiber B. M. Farmer S. R. (2005). PPARgamma2 regulates lipogenesis and lipid accumulation in steatotic hepatocytes. Am. J. Physiol. Endocrinol. Metab. 288, E1195–E1205. 10.1152/ajpendo.00513.2004 15644454

[B88] Schmeisser K. Mansfeld J. Kuhlow D. Weimer S. Priebe S. Heiland I.. (2013). Role of sirtuins in lifespan regulation is linked to methylation of nicotinamide. Nat. Chem. Biol. 9, 693–700. 10.1038/nchembio.1352 24077178 PMC4076143

[B89] Schrauwen-Hinderling V. B. Schrauwen P. Hesselink M. K. C. Van Engelshoven J. M. A. Nicolay K. Saris W. H. M.. (2003). The increase in intramyocellular lipid content is a very early response to training. J. Clin. Endocr. Metab. 88, 1610–1616. 10.1210/jc.2002-021464 12679446

[B90] Schweiger M. Schreiber R. Haemmerle G. Lass A. Fledelius C. Jacobsen P.. (2006). Adipose triglyceride lipase and hormone-sensitive lipase are the major enzymes in adipose tissue triacylglycerol catabolism. J. Biol. Chem. 281, 40236–40241. 10.1074/jbc.M608048200 17074755

[B91] Schwer B. Verdin E. (2008). Conserved metabolic regulatory functions of sirtuins. Cell Metab. 7, 104–112. 10.1016/j.cmet.2007.11.006 18249170

[B92] Sharples A. P. Hughesb D. C. Deane C. S. Saini A. Selman C. Stewart C. E. (2015). Longevity and skeletal muscle mass: the role of IGF signalling, the sirtuins, dietary restriction and protein intake. Aging cell 14, 511–523. 10.1111/acel.12342 25866088 PMC4531066

[B93] Shimizu M. Swanson P. Fukada H. Hara A. Dickhoff W. W. (2000). Comparison of extraction methods and assay validation for salmon insulinlike growth factor-I using commercially available components. Gen. Comp. Endocrinol. 119, 26–36. 10.1006/gcen.2000.7498 10882546

[B94] Shimizu N. Maruyama T. Yoshikawa N. Matsumiya R. Ma Y. Ito N. (2015). A muscle-liver-fat signalling axis is essential for central control of adaptive adipose remodeling. Nat. Commun. 6:6693 10.1038/ncomms7693 25827749 PMC4396397

[B95] Silbernagel G. Kovarova M. Cegan A. Machann J. Schick F. Lehmann R.. (2012). High hepatic SCD1 activity is associated with low liver fat content in healthy subjects under a lipogenic diet. J. Clin. Endocrinol. Metab. 97, E2288–E2292. 10.1210/jc.2012-2152 23015656

[B96] Simó-Mirabet P. Bermejo-Nogales A. Calduch-Giner J. A. Pérez-Sánchez J. (2017a). Tissue-specific gene expression and fasting regulation of sirtuin family in gilthead sea bream (*Sparus aurata*). J. Comp. Physiol. B Biochem. Syst. Environ. Physiol. 187, 153–163. 10.1007/s00360-016-1014-0 27431591

[B97] Simó-Mirabet P. Piazzon M. C. Calduch-Giner J. A. Ortiz Á. Puyalto M. Sitjà-Bobadilla A.. (2017b). Sodium salt medium-chain fatty acids and Bacillus-based probiotic strategies to improve growth and intestinal health of gilthead sea bream (*Sparus aurata*). PeerJ 5:e4001. 10.7717/peerj.4001 29226031 PMC5719961

[B98] Srivastava S. Diaz F. Iommarini L. Aure K. Lombes A. Moraes C. T. (2009). PGC-1a/b induced expression partially compensates for respiratory chain defects in cells from patients with mitochondrial disorders. Hum. Mol. Genet. 18, 1805–1812. 10.1093/hmg/ddp093 19297390 PMC2722224

[B99] Stanton D. A. Alway S. E. Mohamed J. S. (2017). The role of Sirtuin 2 in the regulation of myogenesis. FASEB J. 31(1 Suppl.), 877.13–877.13. 10.1096/fasebj.31.1_supplement.877.13

[B100] Tannahill G. M. (2013). Succinate is an inflammatory signal that induces IL-1 through HIF-1 α. Nature 496, 238–242. 10.1038/nature11986 23535595 PMC4031686

[B101] Tarantino G. Finelli C. Scopacasa F. Pasanisi F. Contaldo F. Capone D.. (2014). Circulating levels of sirtuin 4, a potential marker of oxidative metabolism, related to coronary artery disease in obese patients suffering from NAFLD, with normal or slightly increased liver enzymes. Oxid. Med. Cell. Longev. 2014:920676. 10.1155/2014/920676 25045415 PMC4086623

[B102] Teigen L. E. Orczewska J. I. McLaughlin J. O'Brien K. M. (2015). Cold acclimation increases levels of some heat shock protein and sirtuin isoforms in threespine stickleback. Comp. Biochem. Physiol. A Mol. Integr. Physiol. 188, 139–147. 10.1016/j.cbpa.2015.06.028 26123780

[B103] Tripathy S. Lytle K. A. Stevens R. D. Bain J. R. Newgard C. B. Greenberg A. S.. (2014). Fatty acid elongase-5 (Elovl5) regulates hepatic triglyceride catabolism in obese C57BL/6J mice. J. Lipid Res. 55, 1448–1464. 10.1194/jlr.M050062 24814977 PMC4076069

[B104] Vakhrusheva O. Smolka C. Gajawada P. Kostin S. Boettger T. Kubin T.. (2008). Sirt7 increases stress resistance of cardiomyocytes and prevents apoptosis and inflammatory cardiomyopathy in mice. Circ. Res. 102, 703–710. 10.1161/CIRCRESAHA.107.164558 18239138

[B105] de Celis L. S. V. Gómez-Requeni P. Pérez-Sánchez J. (2004). Production and characterization of recombinantly derived peptides and antibodies for accurate determinations of somatolactin, growth hormone and insulin-like growth factor-I in European sea bass (*Dicentrarchus labrax*). Gen. Comp. Endocrinol. 139, 266–277. 10.1016/j.ygcen.2004.09.017 15560873

[B106] Vélez E. J. Azizi S. Millán-Cubillo A. Fernández-Borràs J. Blasco J. Chan S. J.. (2016). Effects of sustained exercise on GH-IGFs axis in gilthead sea bream (*Sparus aurata*). Am. J. Physiol. Regul. Integr.Comp. Physiol. 310, R313–R322. 10.1152/ajpregu.00230.2015 26661095

[B107] Verdin E. Hirschey M. D. Finley L. W. Haigis M. C. (2010). Sirtuin regulation of mitochondria: energy production, apoptosis, and signaling. Trends Biochem. Sci. 35, 669–675. 10.1016/j.tibs.2010.07.003 20863707 PMC2992946

[B108] Wahli W. Michalik L. (2012). PPARs at the crossroads of lipid signaling and inflammation. Trends Endocrinol. Metab. 23, 351–363. 10.1016/j.tem.2012.05.001 22704720

[B109] Wang F. Wang K. Xu W. Zhao S. Ye D. Wang. (2017). SIRT5 desuccinylates and activates pyruvate kinase M2 to block macrophage IL-1β production and to prevent DSS-induced colitis in mice. Cell Rep. 19, 2331–2344. 10.1016/j.celrep.2017.05.065 28614718

[B110] Wang X. Buechler N. L. Martin A. Wells J. Yoza B. McCall C. E. (2016). Sirtuin-2 regulates sepsis inflammation in ob/ob mice. PLoS ONE 11:e0160431 10.1371/journal.pone.0160431 27500833 PMC4976857

[B111] Wellen K. E. Thompson C. B. (2010). Cellular metabolic stress: considering how cells respond to nutrient excess. Mol. Cell 40, 323–332. 10.1016/j.molcel.2010.10.004 20965425 PMC3190402

[B112] Wenz T. (2013). Regulation of mitochondrial biogenesis and PGC-1a under cellular stress. Mitochondrion 13, 134–142. 10.1016/j.mito.2013.01.006 23347985

[B113] Wu G. Song C. Lu H. Jia L. Yang G. Shi X.. (2014). Sirt2 induces C2C12 myoblasts proliferation by activation of the ERK1/2 pathway. Acta Biochim. Biophys. Sin. 46, 342–345. 10.1093/abbs/gmt151 24457518

[B114] Yúfera M. Perera E. Mata-Sotres J. A. Calduch-Giner J. Martínez-Rodríguez G. Pérez-Sánchez J. (2017). The circadian transcriptome of marine fish (*Sparus aurata*) larvae reveals highly synchronized biological processes at the whole organism level. Sci. Rep. 7:12943. 10.1038/s41598-017-13514-w 29021622 PMC5636797

[B115] Zhang Y. A. Salinas I. Li J. Parra D. Bjork S. Xu Z.. (2010). IgT, a primitive immunoglobulin class specialized in mucosal immunity. Nat. Immunol. 11, 827–835. 10.1038/ni.1913 20676094 PMC3459821

[B116] Zhao S. Xu W. Jiang W. Yu W. Lin Y. Zhang T.. (2010). Regulation of cellular metabolism by protein lysine acetylation. Science 327, 1000–1004. 10.1126/science.1179689 20167786 PMC3232675

