# Supplementary material for: Co-expression Analysis of Sirtuins and Related Metabolic Biomarkers in Juveniles of Gilthead Sea Bream (Sparus aurata) With Differences in Growth Performance
Source: Front Physiol. 2018 Jun 5;9:608. doi: 10.3389/fphys.2018.00608 (PMC5996159; doi:10.3389/fphys.2018.00608)
Supplement: Supplementary file 1 [file Table_1.docx]

| **LOCUS** | **Locus-1** | | **Locus-2** | | **Locus-3** | | **Locus-4** | | **Locus-5** | | **Locus-6** | | **Locus-7** | | **Locus-8** | | **Locus-9** | | **Locus-10** | |  | |
| --- | --- | --- | --- | --- | --- | --- | --- | --- | --- | --- | --- | --- | --- | --- | --- | --- | --- | --- | --- | --- | --- | --- |
| **Internal Code** | L11 | | E4 | | A5 | | M5 | | F6 | | I9 | | C12 | | C3 | | D4 | | E1 | | **AVERAGE** | |
| **Linkage group** | 10 | | 11 | | 20 | | 24 | | 3 | | 19 | | 18 | | 6 | | 1 | | 23 | |  |  |
| **Population**  **(Strain)** | **1** | **2** | **1** | **2** | **1** | **2** | **1** | **2** | **1** | **2** | **1** | **2** | **1** | **2** | **1** | **2** | **1** | **2** | **1** | **2** | **1** | **2** |
| **Heterocigocity observed** | 0.67 | 0.85 | 1.00 | 0.85 | 0.60 | 0.38 | 0.70 | 0.88 | 0.62 | 0.62 | 0.86 | 0.77 | 0.40 | 0.42 | 0.97 | 0.81 | 0.60 | 0.55 | 0.83 | 0.92 | 0.72 | 0.70 |
| **Chi^2^-Pearson** | 12.59 | | 74 | | 58.28 | | 361.67 | | 100.11 | | 67.38 | | 197.29 | | 129.78 | | 98.89 | | 68 | | 1,517.26 | |
| **Significance** | 0.083 | | 0 | | 0 | | 0 | | 0 | | 0 | | 0 | | 0 | | 0 | | 0 | | **0** | |
| **Locus-NAME** | Dld-16-F | | Bld-18-F | | Bt-14-F | | Ct27 | | Hd-25-F | | At37 | | Dt47 | | Eid-39-T | | P3 | | Bd-68-T | | **Strain 1** | **Strain 2** |
| **No. Alleles** | 4 | 5 | 8 | 8 | 4 | 3 | 10 | 13 | 5 | 6 | 5 | 6 | 4 | 5 | 16 | 17 | 7 | 8 | 5 | 5 | 6.8 | 7.6 |

**Supplementary Table 1.** Genetic diversity in strains 1 and 2 by using SMsa1 (Lee-Montero et al., 2013).
