# Supplementary material for: Co-expression Analysis of Sirtuins and Related Metabolic Biomarkers in Juveniles of Gilthead Sea Bream (Sparus aurata) With Differences in Growth Performance
Source: Front Physiol. 2018 Jun 5;9:608. doi: 10.3389/fphys.2018.00608 (PMC5996159; doi:10.3389/fphys.2018.00608)
Supplement: Supplementary file 2 [file Table_2.docx]

**Supplementary Table 2.** Forward and reverse primers of the liver/white skeletal muscle/adipose tissue pathway-focused PCR array.

| Gene name | Symbol |  | Primer sequence |
| --- | --- | --- | --- |
|  |  |  |  |
| Sirtuin1 | *sirt1* | F | GGT TCC TAC AGT TTC ATC CAG CAG CAC ATC |
|  |  | R | CCT CAG AAT GGT CCT CGG ATC GGT CTC |
|  |  |  |  |
| Sirtuin2 | *sirt2* | F | GAA CAA TCC GAC GAC AGC AGT GAA G |
|  |  | R | AGG TTA CGC AGG AAG TCC ATC TCT |
|  |  |  |  |
| Sirtuin3 | *sirt3* | F | CTG CCA AGT CCT CAT CCC |
|  |  | R | CTT CAC CAG ACG AGC CAC |
|  |  |  |  |
| Sirtuin4 | *sirt4* | F | GGC TGG CGG AGT CGG ATG |
|  |  | R | TCC TGA ATA CAC CTG TGA CGA AGA C |
|  |  |  |  |
| Sirtuin5 | *sirt5* | F | CAG ACA TCC TAA CCC GAG CAG AG |
|  |  | R | CCA CGA GGC AGA GGT CAC A |
|  |  |  |  |
| Sirtuin6 | *sirt6* | F | ACT CCA CCA CCA CCG ATG TCA A |
|  |  | R | CTC CTC CTC CTT CAC CTT TCG CTT TG |
|  |  |  |  |
| Sirtuin7 | *sirt7* | F | CTG GAG CAA CCT CTA AAC TGG AA |
|  |  | R | CAC CTT CAG ACT GGA GCC TAA |
|  |  |  |  |
| Proliferator-activated receptor gamma coactivator 1 alpha | *pgc1α* | F | CGT GGG ACA GGT GTA ACC AGG ACT C |
|  |  | R | ACC AAC CAA GGC AGC ACA CTC TAA TTC T |
|  | |  |  |
| Citrate synthase | *cs* | F | TCC AGG AGG TGA CGA GCC |
|  |  | R | GTG ACC AGC AGC CAG AAG AG |
|  |  |  |  |
| NADH-ubiquinone oxidoreductase chain 2 | *nd2* | F | TAG GTT GAA TGA CCA TCG TA |
|  |  | R | GGC TAA GGA GTT GAG GTT |
|  |  |  |  |
| Cytochrome c oxidase subunit I | *cox1* | F | GTC CTA CTT CTT CTG TCC CTT CCT GTT CT |
|  |  | R | AGG TTT CGG TCT GTA AGG AGC ATT GTA ATC |
|  |  |  |  |
| Uncoupling protein1 | *ucp1* | F | GCA CAC TAC CCA ACA TCA CAA G |
|  |  | R | CGC CGA ACG CAG AAA CAA AG |
|  |  |  |  |
| Uncoupling protein2 | *ucp2* | F | CGG CGG CGT CCT CAG TTG |
|  |  | R | AAG CAA GTG GTC CCT CTT TGG TCA T |
|  |  |  |  |
| Uncoupling protein3 | *ucp3* | F | AGG TGC GAC TGG CTG ACG |
|  |  | R | TTC GGC ATA CAA CCT CTC CAA AG |
|  |  |  |  |
| Peroxisome proliferator-activated receptor α | *pparα* | F | TCT CTT CAG CCC ACC ATC CC |
|  |  | R | ATC CCA GCG TGT CGT CTC C |
|  |  |  |  |
| Peroxisome proliferator-activated receptor γ | *pparγ* | F | CGC CGT GGA CCT GTC AGA GC |
|  |  | R | GGA ATG GAT GGA GGA GGA GGA GAT GG |
|  |  |  |  |
| Elongation of very long chain fatty acids 4 | *elovl4* | F | CGG TGG CAA TCA TCT TCC |
|  |  | R | TCA ACT GGC TGT CTG TGT |
|  |  |  |  |
| Elongation of very long chain fatty acids 5 | *elovl5* | F | CCT CCT GGT GCT CT ACA AT |
|  |  | R | GTG AGT GTC CTG GCA GTA |
|  |  |  |  |
| Elongation of very long chain fatty acids 6 | *elovl6* | F | GTG CTG CTC TAC TCC TGG TA |
|  |  | R | ACG GCA TGG ACC AAG TAG T |
|  |  |  |  |
| Fatty acid desaturase 2 | *fads2* | F | GCA GGC GGA GAG CGA CGG TCT GTT CC |
|  |  | R | AGC AGG ATG TGA CCC AGG TGG AGG CAG AAG |
|  |  |  |  |
| Stearoyl-CoA desaturase 1a | *scd1a* | F | CGG AGG CGG AGG CGT TGG AGA AGA AG |
|  |  | R | AGG GAG ACG GCG TAC AGG GCA CCT ATA TG |
|  |  |  |  |
| Stearoyl-CoA desaturase 1b | *scd1b* | F | GCT CAA TCT CAC CAC CGC CTT CAT AG |
|  |  | R | GCT GCC GTC GCC CGT TCT CTG |
|  |  |  |  |
| Phosphatidylethanolamine N-methyltransferase | *pemt* | F | TTG GTG CCA GTC CTG TTG GTC TC |
|  |  | R | TGA TAG ATC AGT CCA GTG AAT GGT CCT TC |
|  |  |  |  |
| Hepatic lipase | *hl* | F | TTG TAG AAG GTG AGG AAA ACT G |
|  |  | R | GCT CTC CAT CAG ACC ATC C |
|  |  |  |  |
| Lipoprotein lipase | *lpl* | F | CGT TGC CAA GTT TGT GAC CTG |
|  |  | R | AGG GTG TTC TGG TTG TCT GC |
|  |  |  |  |
| Hormone sensitive lipase | *hsl* | F | GCT TTG CTT CAG TTT ACC ACC ATT TC |
|  |  | R | GAT GTA GCG ACC CTT CTG GAT GAT GTG |
|  |  |  |  |
| Adipose triglyceride lipase | *atgl* | F | GTG CTT CAG TCC TGG ATG TCT TC |
|  |  | R | AGC CTT GCA GGT CCA TGT TGA |
|  |  |  |  |
| β-Actin | *actb* | F | TCCTGCGGAATCCATGAGA |
|  |  | R | GACGTCGCACTTCATGATGCT |
