# Supplementary material for: Co-expression Analysis of Sirtuins and Related Metabolic Biomarkers in Juveniles of Gilthead Sea Bream (Sparus aurata) With Differences in Growth Performance
Source: Front Physiol. 2018 Jun 5;9:608. doi: 10.3389/fphys.2018.00608 (PMC5996159; doi:10.3389/fphys.2018.00608)
Supplement: Supplementary file 3 [file Table_3.DOCX]

**Supplementary Table 3.**  Forward and reverse primers of the intestine tissue pathway-focused PCR array.

| Gene name | | | Symbol | | | | |  | | Primer sequence | | | | |  | |  |  |
| --- | --- | --- | --- | --- | --- | --- | --- | --- | --- | --- | --- | --- | --- | --- | --- | --- | --- | --- |
|  | |  | | | | |  | | | |  |  | |  | |  |  |  |
| Sirtuin1 | | *sirt1* | | | | | F | | | | GGT TCC TAC AGT TTC ATC CAG CAG CAC ATC | | | | | | |  |
|  |  |  |  |  |  |  | R | | | | CCT CAG AAT GGT CCT CGG ATC GGT CTC | | | | | | |  |
|  | |  | | | | |  | | | |  |  | |  | |  | |  |
| Sirtuin2 | | *sirt2* | | | | | F | | | | GAA CAA TCC GAC GAC AGC AGT GAA G | | | | | | |  |
|  |  |  |  |  |  |  | R | | | | AGG TTA CGC AGG AAG TCC ATC TCT | | | | |  |  |  |
|  | |  | | | | |  | | | |  |  | |  | |  |  |  |
| Sirtuin3 | | *sirt3* | | | | | F | | | | CTG CCA AGT CCT CAT CCC | | | | |  |  |  |
|  |  |  |  |  |  |  | R | | | | CTT CAC CAG ACG AGC CAC | | | | |  |  |  |
|  | |  | | | | |  | | | |  |  | |  | |  |  |  |
| Sirtuin4 | | *sirt4* | | | | | F | | | | GGC TGG CGG AGT CGG ATG | | | | |  |  |  |
|  |  |  |  |  |  |  | R | | | | TCC TGA ATA CAC CTG TGA CGA AGA C | | | | | | |  |
|  | |  | | | | |  | | | |  |  | |  | |  | |  |
| Sirtuin5 | | *sirt5* | | | | | F | | | | CAG ACA TCC TAA CCC GAG CAG AG | | | | |  | |  |
|  |  |  |  |  |  |  | R | | | | CCA CGA GGC AGA GGT CAC A | | | | |  | |  |
|  | |  | | | | |  | | | |  |  | |  | |  | |  |
| Sirtuin6 | | *sirt6* | | | | | F | | | | ACT CCA CCA CCA CCG ATG TCA A | | | | |  | |  |
|  |  |  |  |  |  |  | R | | | | CTC CTC CTC CTT CAC CTT TCG CTT TG | | | | |  | |  |
|  | |  | | | | |  | | | |  |  | |  | |  | |  |
| Sirtuin7 | | *sirt7* | | | | | F | | | | CTG GAG CAA CCT CTA AAC TGG AA | | | | |  | |  |
|  |  |  |  |  |  |  | R | | | | CAC CTT CAG ACT GGA GCC TAA | | | | |  | |  |
|  |  |  |  |  |  |  |  |  |  |  |  |  |  |  |  |  |  |  |
| Proliferator-activated receptor gamma coactivator 1 alpha | | *pgc1α* | | | | | F | | | | CGT GGG ACA GGT GTA ACC AGG ACT C | | | | |  | |  |
|  |  |  | | | | | R | | | | ACC AAC CAA GGC AGC ACA CTC TAA TTC T | | | | |  | |  |
|  | |  | | | | |  | | | |  | | | | |  | |  |
| Carnitine palmitoyltransferase 1A | | *cpt1a* | | | | | F | | | | GTG CCT TCG TTC GTT CCA TGA TC | | | | |  | |  |
|  |  |  |  |  |  |  | R | | | | TGA TGC TTA TCT GCT GCC TGT TTG | | | | |  | |  |
|  | |  | | | | |  | | | |  |  | |  | |  | |  |
| Citrate synthase | | *cs* | | | | | F | | | | TCC AGG AGG TGA CGA GCC | | | | |  | |  |
|  |  |  |  |  |  |  | R | | | | GTG ACC AGC AGC CAG AAG AG | | | | |  | |  |
|  | |  | | | | |  | | | |  |  | |  | |  | |  |
| NADH-ubiquinone oxidoreductase chain 2 | | *nd2* | | | | | F | | | | TAG GTT GAA TGA CCA TCG TA | | | | |  | |  |
|  |  |  |  |  |  |  | R | | | | GGC TAA GGA GTT GAG GTT | | | | |  | |  |
|  | |  | | | | |  | | | |  |  | |  | |  | |  |
| Cytochrome c oxidase subunit I | | *cox1* | | | | | F | | | | GTC CTA CTT CTT CTG TCC CTT CCT GTT CT | | | | | | |  |
|  |  |  |  |  |  |  | R | | | | AGG TTT CGG TCT GTA AGG AGC ATT GTA ATC | | | | | | |  |
|  | |  | | | | |  | | | |  |  | |  | |  | |  |
| Uncoupling protein1 | | *ucp1* | | | | | F | | | | GCA CAC TAC CCA ACA TCA CAA G | | | | |  | |  |
|  |  |  |  |  |  |  | R | | | | CGC CGA ACG CAG AAA CAA AG | | | | |  | |  |
|  | |  | | | | |  | | | |  | | | | |  | |  |
| Proliferating cell nuclear antigen | | *pcna* | | | | | F | | | | CGT ATC TGC CGT GAC CTG T | | | | |  | |  |
|  |  |  |  |  |  |  | R | | | | AGA ACT TGA CTC CGT CCT TGG | | | | | | |  |
|  | |  | |  | | | | |  |  | | | | | | |  | |
| Occludin | | *ocln* | | | | | F | | | | GTG TCA GAA CCT CTA CCA GAC CAG CTA CTC | | | | | | |  |
|  |  |  |  |  |  |  | R | | | | GAA AGC CTC CCA CTC CTC CCA TCT | | | | | | |  |
|  | |  | | | | |  | | | |  | | | | | | |  |
| Cadherin-1 | | *cdh1* | | | | | F | | | | TGC TCC ATA CAG CGT CAC CTT ACA | | | | |  | |  |
|  |  |  |  |  |  |  | R | | | | CTC GTT CAT CCT AGC CGT CCA GTT | | | | |  | |  |
|  | |  | | | | |  | | | |  |  | |  | |  | |  |
| Cadherin-17 | | *cdh17* | | | | | F | | | | GAT GCC CGC AAC CCA GAG | | | | |  | |  |
|  |  |  |  |  |  |  | R | | | | CCG TTG ATT CAC TGC CGT AGA C | | | | |  | |  |
|  | |  | | | | |  | | | |  |  | |  | |  | |  |
| Intestinal-type alkaline phosphatase | | *alpi* | | | | | F | | | | CCG CTA TGA GTT GGA CCG TGA T | | | | |  | |  |
|  |  |  |  |  |  |  | R | | | | GCT TTC TCC ACC ATC TCA GTA AGG G | | | | |  | |  |
|  | |  | | | | |  | | | |  |  | |  | |  | |  |
| Intestinal fatty acid-binding protein | | *fabp2* | | | | | F | | | | CGA GCA CAT TCC GCA CCA AAG | | | | |  | |  |
|  |  |  |  |  |  |  | R | | | | CCC ACG CAC CCG AGA CTT C | | | | |  | |  |
|  | |  | | | | |  | | | |  |  | |  | |  | |  |
| Ileal fatty acid-binding protein | | *fabp6* | | | | | F | | | | ACC CAG GAC GGC AAT ACC | | | | |  | |  |
|  |  |  |  |  |  |  | R | | | | CGA CGG TGA AGT TGT TGG T | | | | |  | |  |
|  | |  | | | | |  | | | |  |  | |  | |  | |  |
| Mucin 2 | | *muc2* | | | | | F | | | | ACG CTT CAG CAA TCG CAC CAT | | | | |  | |  |
|  |  |  |  |  |  |  | R | | | | CCA CAA CCA CAC TCC TCC ACA T | | | | |  | |  |
|  | |  | | | | |  | | | |  |  | |  | |  | |  |
| Mucin 13 | | *muc13* | | | | | F | | | | TTC AAA CCC GTG TGG TCC AG | | | | |  | |  |
|  |  |  |  |  |  |  | R | | | | GCA CAA GCA GAC ATA GTT CGG ATA T | | | | | | |  |
|  | | |  | |  | | | |  |  | |  | |  | |  |  |  |
| Transcription factor HES-1-B | | *hes1-b* | | | | | F | | | | GCC TGC CGA TAT GAT GGA A | | | | |  | |  |
|  |  |  |  |  |  |  | R | | | | GGA GTT GTG TTC ATG CTT GC | | | | |  | |  |
|  | |  | | | | |  | | | |  |  | |  | |  | |  |
| Tumor necrosis factor-alpha | | *tnfα* | | | | | F | | | | CAG GCG TCG TTC AGA GTC TC | | | | |  | |  |
|  |  |  |  |  |  |  | R | | | | CTG TGG CTG AGA GCT GTG AG | | | | |  | |  |
|  | |  | | | | |  | | | |  |  | |  | |  | |  |
| Interleukin-1 beta | | *il1β* | | | | | F | | | | GCG ACC TAC CTG CCA CCT ACA CC | | | | |  | |  |
|  |  |  |  |  |  |  | R | | | | TCG TCC ACC GCC TCC AGA TGC | | | | |  | |  |
|  | |  | | | | |  | | | |  |  | |  | |  | |  |
| Interleukin-6 | | *il6* | | | | | F | | | | TCT TGA AGG TGG TGC TGG AAG TG | | | | |  | |  |
|  |  |  |  |  |  |  | R | | | | AAG GAC AAT CTG CTG GAA GTG AGG | | | | |  | |  |
|  | |  | | | | |  | | | |  |  | |  | |  | |  |
| Interleukin-10 | | *il10* | | | | | F | | | | AAC ATC CTG GGC TTC TAT CTG | | | | |  | |  |
|  |  |  |  |  |  |  | R | | | | GTG TCC TCC GTC TCA TCT G | | | | |  | |  |
|  | |  | | | | |  | | | |  |  | |  | |  | |  |
| CD4 | | *cd4* | | | | | F | | | | TCC TCC TCC TCG TCC TCG TT | | | | |  | |  |
|  |  |  |  |  |  |  | R | | | | GGTGTCTCATCTTCCGCTGTCT | | | | |  |  |  |
|  | |  | | | | |  | | | |  |  | |  | |  | |  |
| CD8 alpha | | *cd8a* | | | | | F | | | | GCA GCA ACG GTA ACA CGA ACG | | | | |  |  |  |
|  |  |  |  |  |  |  | R | | | | CCAGTATGAGCGGAGTACAGAACA | | | | |  |  |  |
|  | |  | | | | |  | | | |  |  | |  | |  | |  |
| CD8 beta | | *cd8b* | | | | | F | | | | CCG AAA TGT GGA AGA CTG GAA CTC | | | | |  | |  |
|  |  |  |  |  |  |  | R | | | | CTTTGGAGGTAAGGTTGGAGGGAT | | | | |  |  |  |
|  | |  | | | | |  | | | |  |  | |  | |  | |  |
| Galectin-1 | | *lgals1* | | | | | F | | | | GTG TGA GGA GGT CCG TGA TG | | | | |  |  |  |
|  |  |  |  |  |  |  | R | | | | ACT GTA GAG CCG TCC GAT AGG | | | | |  |  |  |
|  | |  | | | | |  | | | |  |  | |  | |  | |  |
| Galectin-8 | | *lgals8* | | | | | F | | | | GGC GGT GAA CGG CGG TCA | | | | |  |  |  |
|  |  |  |  |  |  |  | R | | | | GCT CCA GCT CCA GTC TGT GTT GAT AC | | | | | | |  |
|  | |  | | | | |  | | | |  |  | |  | |  | |  |
| Secreted Immunoglobulin M | | *sIgM* | | | | | F | | | | ACC TCA GCG TCC TTC AGT GTT TAT GAT GCC | | | | | | |  |
|  |  |  |  |  |  |  | R | | | | CAG CGT CGT CGT CAA CAA GCC AAG C | | | | | | |  |
|  | |  | |  | | |  |  | | |  | |  | |  | |  |  |
| Secreted Immunoglobulin T | | *sIgT* | | | | | F | | | | GCT GTC AAG GTG GCC CCA AAA G | | | | |  |  |  |
|  | |  | | | | | R | | | | CAA CAT TCA TGC GAG TTA CCC TTG GC | | | | |  |  |  |
|  | |  | |  | |  | | |  |  | | |  | |  | |  | |
| Membrane Immunoglobulin M | | *mIgM* | | | | | F | | | | GCTATGGAGGCGGAGGAAGATAACA | | | | |  |  |  |
|  |  |  | | | | | R | | | | GCAGAGTGATGAGGAAGAGAAGGATGAA | | | | |  |  |  |
|  | |  | |  | |  | | |  |  | | |  | |  | |  | |
| Membrane Immunoglobulin T | | *mIgT* | | | | | F | | | | AGA CGA TGC CAG TGA AGA GGA TGA GT | | | | |  |  |  |
|  |  |  | | | | | R | | | | CGA AGG AGG AGG CTG TGG ACC A | | | | |  | |  |
|  | |  | |  | |  | | |  |  | | |  | |  | |  | |
| β-Actin | | *actb* | | |  | | F | | | | TCCTGCGGAATCCATGAGA | | | | |  | |  |
|  |  |  | | |  | | R | | | | GACGTCGCACTTCATGATGCT | | | | |  | |  |
